# The Impact of a 1,2,3-Triazole Motif on the Photophysical Behavior of Non-K Tetrasubstituted Pyrene with a Substitution Pattern Providing the Long Axial Symmetry

**DOI:** 10.3390/molecules27134314

**Published:** 2022-07-05

**Authors:** Dawid Zych, Aneta Slodek

**Affiliations:** 1 Faculty of Chemistry, University of Opole, Oleska 48, 45-052 Opole, Poland; 2 Institute of Chemistry, Faculty of Science and Technology, University of Silesia, Szkolna 9, 40-006 Katowice, Poland; aneta.slodek@us.edu.pl

**Keywords:** pyrene derivatives, triazole motif, substitution pattern, experimental study, photophysical properties, theoretical study

## Abstract

1,3,6,8-Tetrasubstituted pyrene derivatives with two types of substituents (4-(2,2-dimethylpropyloxy)pyridine, 1-decyl-1,2,3-triazole, 1-benzyl-1,2,3-triazole, and pyrazole), substituted in such a way that provides the long axial symmetry, are prepared and characterized in the present study. To the best of our knowledge, the pyrene derivative containing the same heteroaryl motif (triazole) but substituted by two various alkyls, straight decyl and benzyl-based side chains (**C**), is reported for the first time. For comparison, compounds with one kind of triazole motif and substituted pyridine or pyrazole groups were prepared (**A** and **B**). The photophysical properties of all molecules were evaluated by thermogravimetric analysis (TGA) and UV-Vis spectroscopy (absorption and emission spectra, quantum yields, and fluorescence lifetimes). The obtained results were compared to analogues substituted at the 1,3,6,8 positions by one kind of substituent and also with all the 1,3,6,8-tetrasubstituted pyrenes reported in the literature substituted by two kinds of substituents with a substitution pattern that provides long axial symmetry. In addition, theoretical studies based on DFT and TD-DFT were performed that supported the interpretation of the experimental results. The photophysical properties of tetrasubstituted pyrene derivatives having triazole units at the 1,8-positions, respectively, and other identical substituents at the 3,6 positions show the dominance of triazole units in the pyrene framework; the dominance is even higher in the case of the substitution of 1,3,6,8 positions by triazoles, but containing two various alkyls.

## 1. Introduction

**Figure 1:**
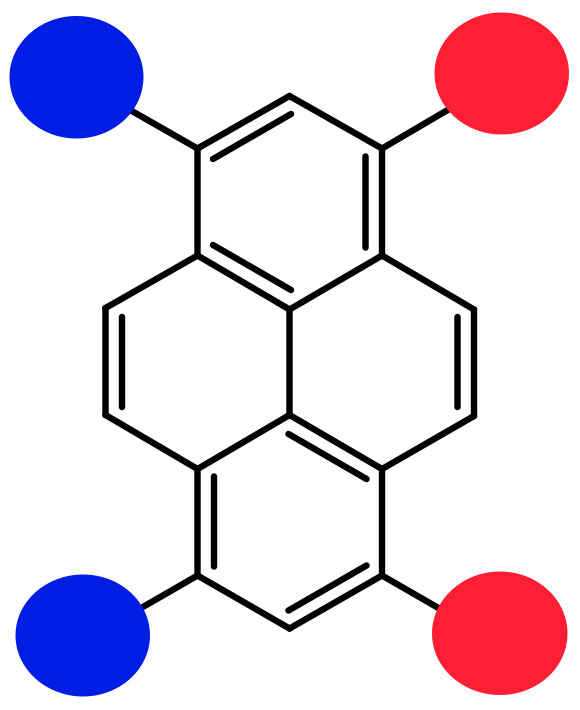
1,3,6,8-Tetrasubstituted pyrene with the substitution pattern providing the long axial symmetry.

In spite of the fact that a long time has passed since the attainment of pyrene in an impure form from coal tar by the French chemist Auguste Laurent (1837) [1] and in pure form by the German chemist Carl Graebe (1870) [2], the design of new effective fluorescent molecules based on the pyrene structure still draws considerable attention from scientists [3,4,5,6]. The many interesting properties of pyrene derivatives, such as efficient fluorescence, long lifetime of the excited state, high quantum yield, the possibility to form the excimers, photostability, thermal stability and a large Stokes shift, make them of considerable interest in the development of fluorophores and sensors for metal ions [7,8,9,10,11]. The increasing progress in the synthetic methods and their accessibility expand the molecules’ spectrum dedicated to material science, medicinal chemistry and molecular biology. The Cu-catalyzed azide-alkyne 1,3-dipolar cycloaddition allows the introduction of triazole moieties into molecules [12]. Incorporating the triazole motif may be an interesting route to tailor the optical properties by coordinating with metal ions. Alireza Khataee et al. reported on pyrene that was modified with a fluorescent anthracene sensor containing the triazole motif for the sensitive detection of iron ions in real samples [13]. To the best of our knowledge, the pyrene derivatives described in the literature include only one kind of substituted group, the 1,2,3-triazole. The other motivation for obtaining appropriately designed pyrene derivatives is their application as NCN-cyclometalating ligands, which have been of interest to our group and the group of Prof. Yu-Wu Zhong for almost 12 years [14,15]. Recently, we presented that the optical properties strongly depend on the substitution pattern; the highest values of fluorescence quantum yields were achieved for 1,3,6,8-tetrasubstituted pyrene with the substitution pattern providing the long axial symmetry (Figure 1) [16,17].

In this paper, taking into account the above-described considerations, we describe the synthesis and study of the properties of novel 1,3,6,8-tetrasubstituted pyrenes containing the triazole motif with the pyridyl (**A**) and pyrazolyl (**B**) groups. Moreover, one of the target pyrenes is substituted only by triazole groups that differ by the substituents at position 1 of the mentioned heteroaryl motif (**C**). The obtained results are compared to analogs substituted at the 1,3,6,8 positions by one kind of substituents and with all the 1,3,6,8-tetrasubstituted pyrenes reported in the literature substituted by two types of substituents with the substitution pattern providing the long axial symmetry. The experimental study is supported by the quantum-chemical calculations based on DFT and TD-DFT methods.

## 2. Results and Discussion

**Scheme 1:**
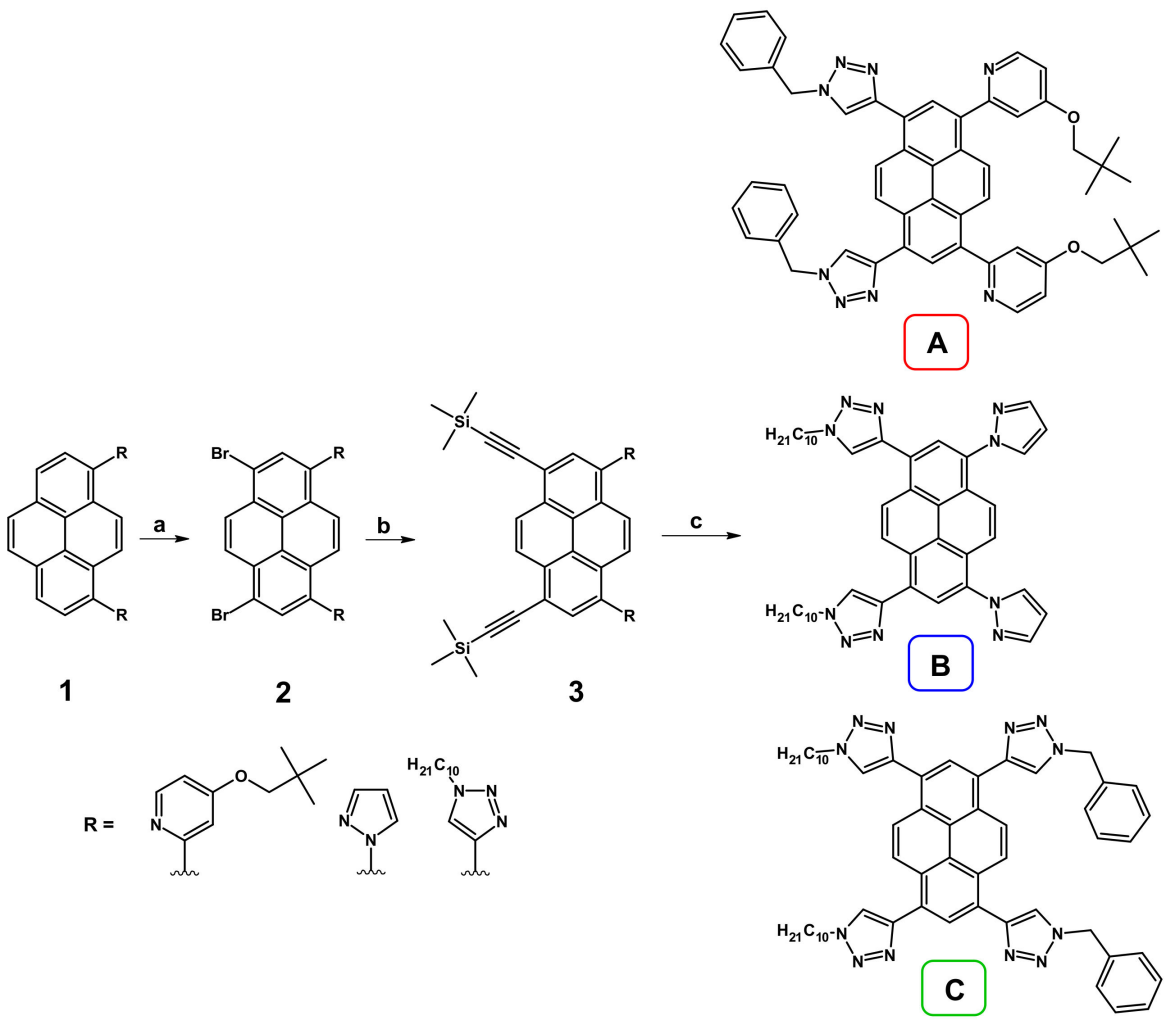
Synthesis of the tetrasubstituted pyrene derivatives **A**–**C**. Reagents and conditions: (**a**) Br_2_, CH_2_Cl_2_, 40 °C, 2 h; (**b**) trimethylsilylacetylene, [Pd(PPh_3_)_4_], CuI, NEt_3_, 1,8-diazabicyclo[5.4.0]undec-7-ene, 90 °C, 16 h; (**c**) appropriate azide, KF, CuSO_4_ 5H_2_O, sodium ascorbate, pyridine, EtOH, H_2_O, room temperature, 24 h.

We previously presented the synthesis of disubstituted pyrenes containing the same heteroaryl groups at positions 1,6- or 1,8- [16,17]. The same procedure was implemented in the current work, which allowed us to obtain of pyrene derivatives with pyridyl, pyrazolyl and triazolyl groups at positions 1 and 8 (Scheme 1). These molecules (**1**) were brominated, resulting in 1,8-dibromo-3,6-di(heteroaryl)pyrenes (**2**). The introduction of (trimethylsilyl)ethynyl groups at positions 1 and 8 was possible by the Sonogashira coupling reaction in the presence of DBU, which resulted in intermediates described as **3**. The last step was based on the Cu-catalyzed 1,3-dipolar cycloaddition, where decyl azide or benzyl azide was used, resulting in the target molecules **A**–**C**.

It is worth emphasizing that, in the case of molecule **C**, two synthetic pathways were checked, the first starting from disubstituted pyrene containing triazoles substituted by decyl groups and the second starting with the analog containing benzyl groups at triazole motifs. A significant difference in the yield of the obtained product was observed. When the substrate with the benzyl substituents was applied, the yield of the last step, 1,3-dipolar cycloaddition, was definitely lower (≈15%) due to the low solubility of the deprotected intermediate in the mixture of solvents (EtOH/H_2_O), which resulted in a compact, almost insoluble solid and slowed down the progress of the reaction.

### 2.1. Theoretical Studies

The DFT (density functional theory) calculations with B3LYP [18] functional with the 6-31G(d,p) basis set implemented in the Gaussian 16 program were conducted [19]. It allowed us to obtain information about the geometry and properties of the molecules. Additionally, the CAM-B3LYP functional [20] was used to calculate the absorption and emission spectra, which proved important to provide more reliable spectra batching and better experimental data with respect to the B3LYP functional [17,21]. All calculations were performed with chloroform as the solvent in the polarizable continuum model (PCM) [22]. All orbitals were computed at an isovalue of 0.025 e/bohr^3^. The B3LYP/6-31G(d,p)-optimized structures of **A**–**C** with contours of the following orbitals HOMO-2, HOMO-1, HOMO, LUMO, LUMO+1 and LUMO+2 and their energies are depicted in Table 1.

**Figure 2:**
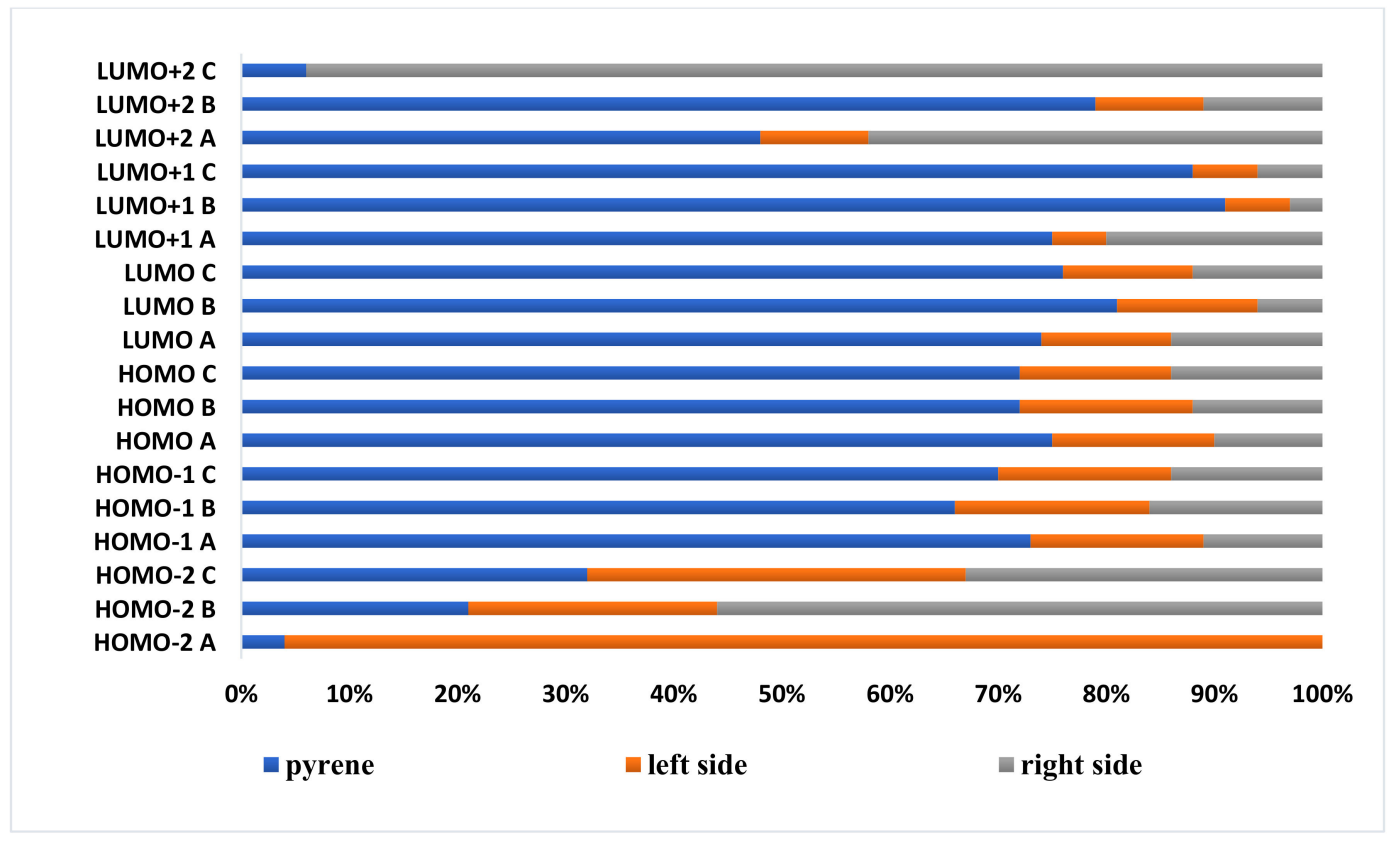
Contribution of the particular parts of the molecules creating orbitals HOMO-2, HOMO-1, HOMO, LUMO, LUMO+1 and LUMO+2 for **A**–**C**.

For a more detailed presentation of the selected orbitals, the contribution of the individual part of molecules **A**–**C** is presented in Figure 2. The molecules were divided into three parts: pyrene, left side and right side. The left side contains, in the case of molecules **A** and **B,** substituted triazole motifs, whereas in the case of molecule **C**, the left side is represented by a triazole motif substituted by a decyl group; the rest of the substituents are described as the right side of the molecules.

The frontier orbitals (HOMO and LUMO) for all molecules **A**–**C** are mainly localized on the pyrene core, which is typical for the pyrene derivatives substituted at the non-K region. In the case of HOMOs, when the substitution by the triazole motif occurs only from one side (**A** (15%)) and (**B** (16%)), the contribution of the triazole in the creation of the mentioned orbital is higher (left side) than the pyridyl (10%) or pyrazolyl (12%) substituents (right side); for compound **C**, containing triazole motifs from both sides of the pyrene core, their contributions do not differ to each other (14%). The same tendency was observed for HOMO-1, whereas the HOMOs-2 of **A**–**C** differ significantly from each other. Taking into account the unoccupied orbitals, in the case of the LUMOs and LUMOs+1 of the target molecules, the same tendency as for HOMO and HOMO-1 was observed for molecule **C**, where the orbital is created in the equal parts by the triazole substituents (12%), whereas in the molecules **A** and **B**, they differ from each other with the higher contribution of triazole for **B** and lower for **A**.

Furthermore, the calculated data allowed us to explain further the molecular properties of the molecules **A**–**C**, such as the chemical hardness (η) (η = 0.5(E_LUMO_ − E_HOMO_) [23], chemical potential (μ) (μ = 0.5(E_LUMO_ + E_HOMO_) [24] and global electrophilicity indexes (ω) (ω = μ^2^/2η) (Table 2) [25].

Global electrophilicity indexes (ω) represent the molecule’s stabilization energy and the molecule’s propensity to accept electrons. These values increase in the following order: **A** < **C** < **B**. The chemical hardness (η) is a parameter that shows the resistance to change in the charge transfer or electron distribution; the highest value was achieved by compound **B**, whereas the lowest was by **C**. The chemical potential (μ) shows the electrons’ escaping tendency from an equilibrium system, which is the same for **A** and **C** and definitely lower for **B**.

**Figure 3:**
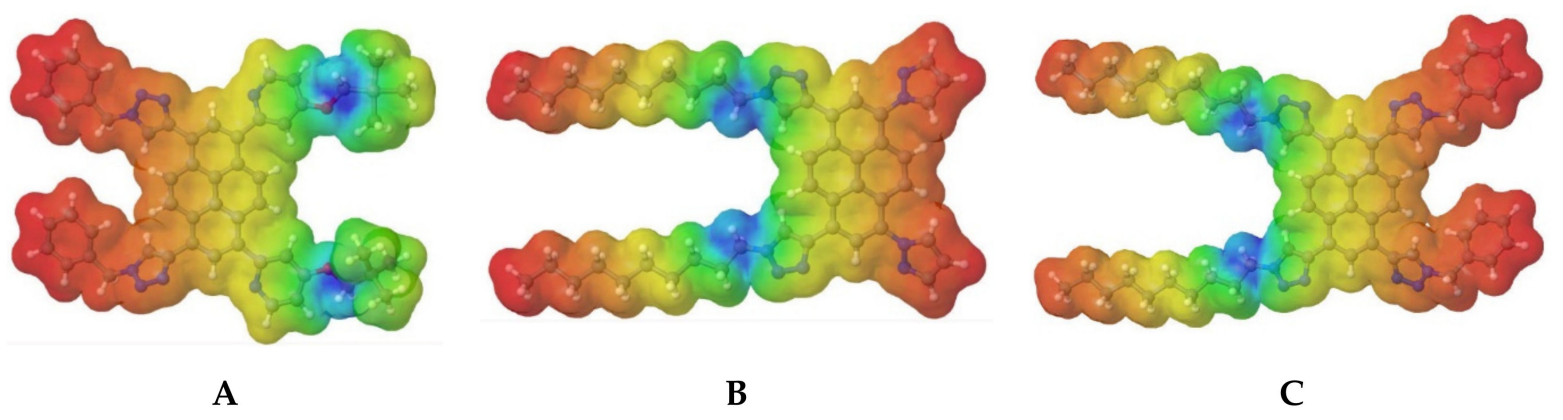
Electrostatic potential energy maps calculated at the B3LYP/6-31G(d,p) level for **A**–**C**.

To understand the chemical reactions and interactions, such as H-bonding, the molecular electrostatic potential maps of molecules **A**–**C** were calculated at the B3LYP/6-31G(d,p) level, and the results are presented in Figure 3.

The molecular electrostatic potential maps describe the nucleophilic and electrophilic sides. The red areas depict the electrophilic activity corresponding to the electron-rich areas, whereas the blue areas indicate nucleophilic activity corresponding to the electron-deficient areas. The results show that triazoles substituted by the decyl group have the highest nucleophilic potential, whereas triazoles substituted by the benzyl group exhibit electron-rich areas.

### 2.2. Thermal Properties

**Figure 4:**
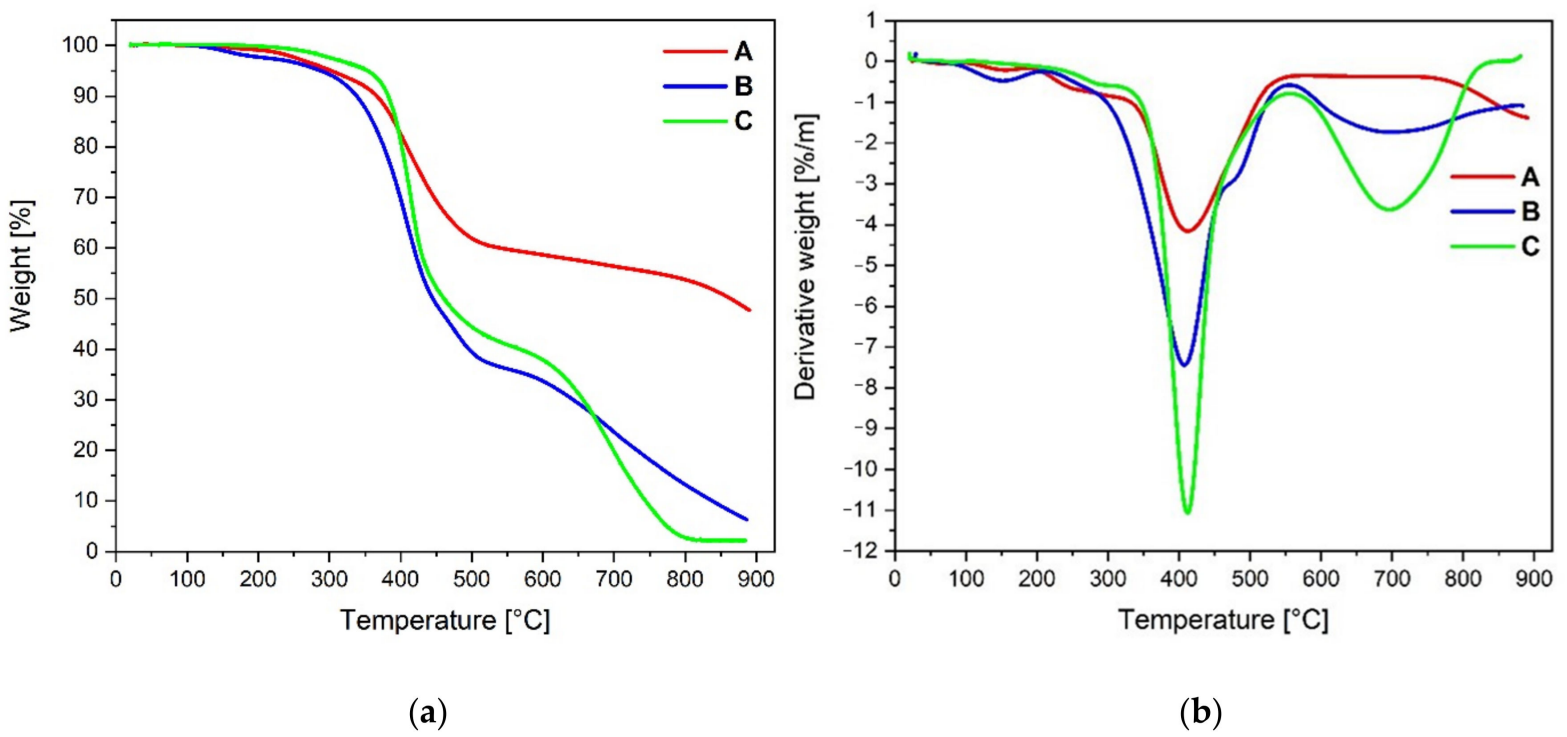
TGA (**a**) and DTG (**b**) curves for **A**–**C**.

The thermal properties of all target molecules **A**–**C** were examined using thermogravimetric analysis in the temperature range up to 900 °C under a nitrogen atmosphere. The obtained data are listed in Table 3, whereas the TGA (a) and DTG (b) spectra are presented in Figure 4.

Among the studied compounds, the highest temperatures corresponding to 5% and 10% weight loss during heating were observed for molecule **C**, at 354 °C and 383 °C, respectively. The least thermally stable was molecule **B**, with temperatures of 288 °C and 338 °C, respectively. Molecules **A** and **C** exhibit three maximal decomposition temperatures, whereas **B** presents four. When comparing the thermal properties of the molecules to the already published analogs containing four identical substituents at positions 1,3,6 and 8 of pyrene (T_5%_, T_10%_), i.e., pyridyl (194 °C, 300 °C), triazolyl with a decyl group (303 °C, 365 °C), triazolyl with a benzyl group (364 °C, 378 °C) [10] and pyrazolyl (294 °C, 305 °C) [16], the values of T_5%_ and T_10%_ obtained for molecules **A**–**C** are mainly between the corresponding values for the 1,3,6,8-tetrasubstituted analogs with the same group of substituents. The highest char residue value was achieved by compound **A** (48%), whereas molecules **B** and **C** show a definitely lower amount of char residue, 6% and 3%, respectively.

### 2.3. Optical Properties and TD-DFT Study

**Figure 5:**
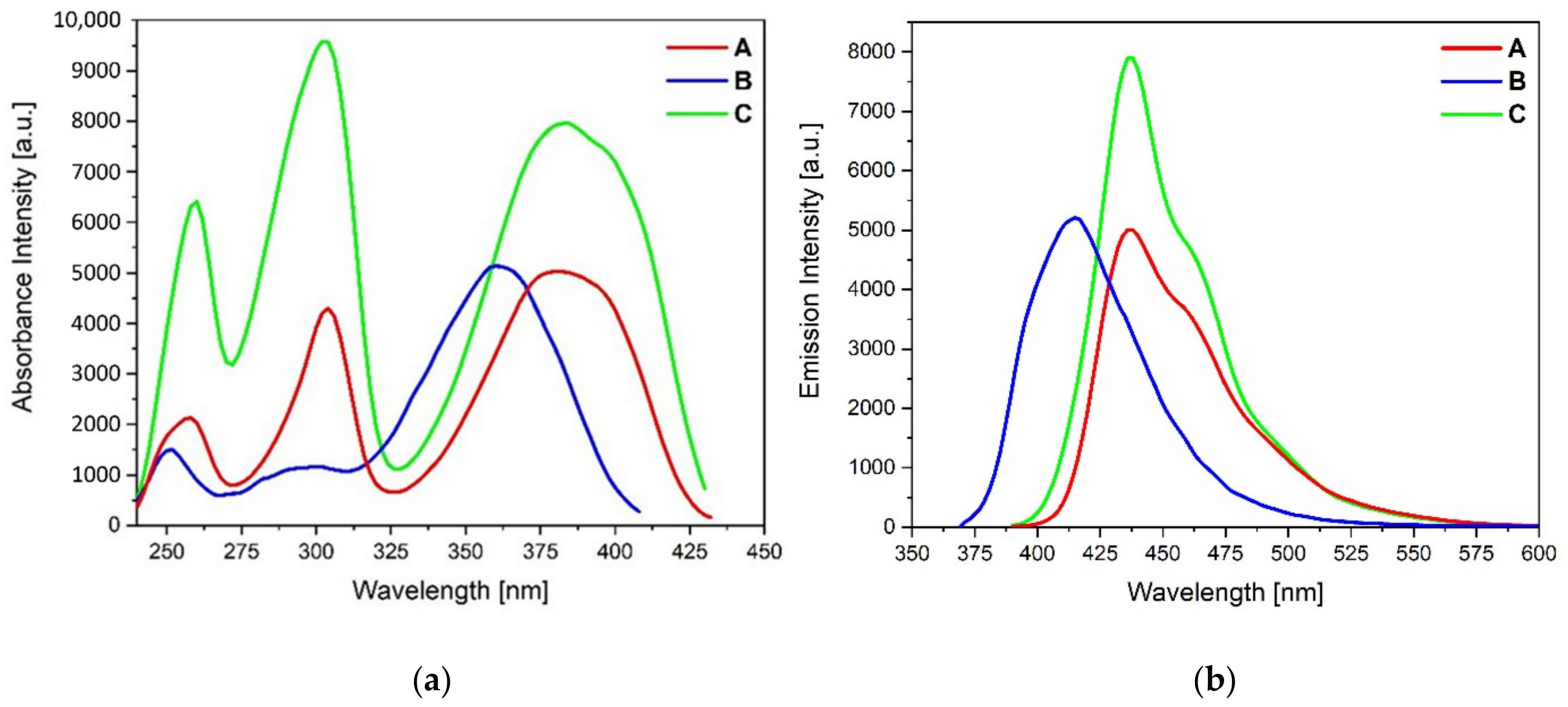
Absorption (**a**) and emission (**b**) spectra recorded in the chloroform solution (*c* = 10^−5^ mol/L) for molecules **A**–**C**.

The spectroscopic parameters of the pyrene derivatives **A**–**C** in chloroform are listed in Table 4, and the absorption and emission spectra are presented in Figure 5. Compounds **A**–**C** display absorption spectra in the range of 250–350 nm, with three well-resolved absorption bands similar to that of the parent pyrene [26]. Compared to the pyrene compounds, **A**–**C** possess broader and red-shifted absorption bands, which is the consequence of the extended conjugation length of the pyrene derivatives **A**–**C** with the substituents at the 1, 3, 6 and 8 positions.

**Figure 6:**
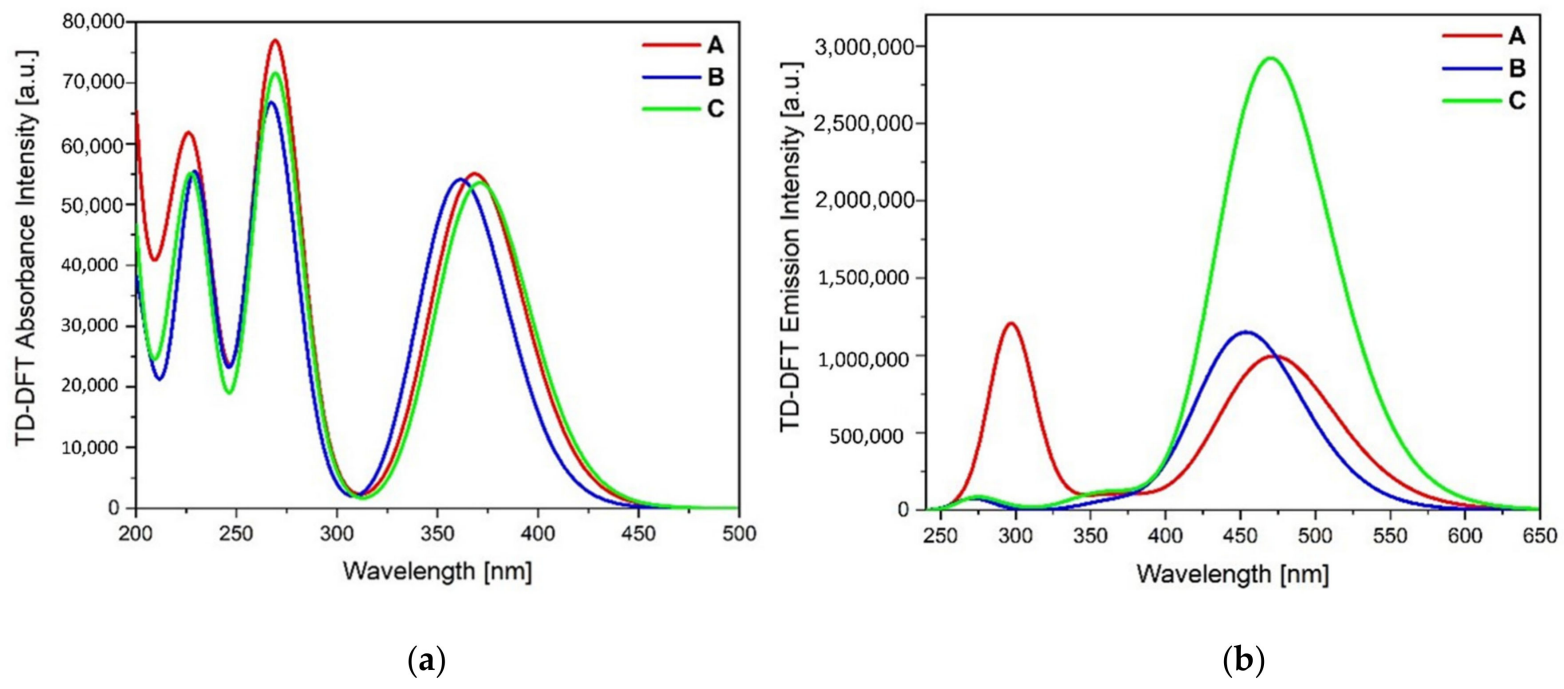
TD-DFT (CAM-B3LYP/6-31G(d,p)) calculated absorption (**a**) and emission (**b**) spectra for molecules **A**–**C**.

Compounds **A** and **C** with the same units of 1-benzyl-1,2,3-triazoles at the 1,8 positions have the absorption maxima at the same wavelength. In contrast, the low energetic band S_0_→S_1_ of compound **B** is shifted to a shorter wavelength of about 30 nm due to the absence of additional substituents at the pyrazolyl units. The experimental spectra confirm this tendency, although the S_0_→S_1_ transition for compound **B** is less blue-shifted, about 10 nm, compared to compounds **A** and **C** (Table 4). The emission spectra of compounds display a bright blue emission at 436 nm for **A**, 415 nm for **B** and 436 nm for **C**, respectively. The emission spectra of **A** and **C** are qualitatively similar and differ primarily in the relative intensities of the vibrational band likely to their absorption spectra. The hyperchromic effect for compound **C** is also confirmed by the calculated absorption and emission spectra by the TD-DFT method (Figure 6; Table 5 and Table 6); the bands and their character were established using GaussSum software [27].

Interestingly, with the same number of substituents but a different pattern, the emission maximum for **A** and **C** was at the same wavelength (*λ*_em_ = 436 nm) and revealed a bathochromic shift of 20 nm compared to compound **B**. These results can be associated with the arrangement of the substituents in the central pyrene moiety. All pyrene derivatives **A**–**C** are symmetrical. Sill, in the case of **A** and **C**, the symmetry is strongly distorted by the arrangement of the decyl chain and benzyl at the triazole units in **C** and benzyl at triazole and neopentyloxy at the pyridyl substituents in **A** and benzyl at the triazole unit (Table 1), causing a bathochromic effect in relation to compound **B** with distortion symmetry only by the decyl alkyl chain in the triazole units at the 1,8 positions. Furthermore, the hyperchromic effect observed in compound **C** can be associated with the long flexible chain at the triazole and the strongest donating character of 1-benzyl-1,2,3-triazole units at the 3,5 positions in **C** compared to the pyridyl substituents in **A** (Figure 2 and Figure 3). The shift of the emission spectra to longer wavelengths for **A** and **C** assigned to lower symmetry and the extended conjugation of π-electron compared to compound **B** indicate that the energy gap between the ground state and excited states decreases. The calculated energy gap for compounds **A** and **C** is the same (E_g opt_ = 2.84 eV) and higher for compound **B** (E_g opt_ = 2.99 eV), and although the estimation of the energy gap by the DFT calculations is slightly higher, it follows the same trend, being comparable for compounds **A** and **C** (E = 3.20 eV for **A** and E = 3.17 eV for **C**) and increasing for **B** (E = 3.25 eV) (Figure 2).

All compounds exhibit high fluorescence quantum yields in the range of 71–81%. The quantum yield for compounds **A** and **C** is very similar, pointing out the exact character of the excited state and higher about 10% than that of compound **B**. Markedly, the fluorescence quantum yield depends on the type of substituents connected with the pyrene molecule. The difference in the quantum yield among **A**–**C** pyrene derivatives is reflected in the radiative process rate constant k_r_, where for the compounds **A** and **C** are four times greater than the non-radiative processes rate constant k_nr,_ while for compound **B**, the k_r_ is only twice bigger than k_nr_ (Table 4). This result indicates a more significant share of non-radiation processes in the deactivation path of compound **B** compared to compounds **A** and **C**, explaining the higher quantum yield of the latter.

This phenomenon is also reflected in the calculated dipole moment (Δμ) tendency, which is the highest for **C** (Table 7).

Although the Δμ values for all **A**–**C** compounds are indeed zero since they possess centrosymmetric symmetry, a minor alteration in the dipole moment upon excitation might appear. The results further indicate that the **A**–**C** should exhibit a weak or no solvatochromic effect, even stressing the soft negative solvatochromic effect (Table 7). This observation is consistent with the slight positive solvatochromic impact shown in the absorption and emission spectra of the tetrasubstituted pyrene derivatives [28,29].

The presence of the triazole units in the presented compounds indicates their role and influence on the photophysical properties of the 1,3,6,8-tetrasubstituted pyrene derivatives. Interestingly, the photophysical properties of the tetrasubstituted pyrene derivatives **1L** and **2L**, having diazole or triazole units at the 1,8 positions, respectively, and the identical bulky pyridinyl substituents in the 3,6 positions, show the influence of the substituents on the 1,8 positions and display the dominance of the triazole units in the pyrene framework (Table 8) [16,17]. Furthermore, when we compared the 1,3,6,8-tetrasubstituted pyrenes **3L**–**5L** (Table 8), having the same diphenylamine units at the 1,8 positions and varying in the type of substituents at the 3,6 positions, the decrease in quantum yield appeared with the increase in the methyl groups in the phenyl substituents at the 3,6 positions without changing the emission maximum [30,31]. However, compounds **6L** and **7L**, differing by the bulky substituents at the 1,8 positions and possessing *tert*-butylphenyl substituents at the 3,6 positions, exhibit a similar quantum yield, whereas the maximum emission for **6L** is red-shifted by about 50 nm concerning **7L**, possibly arising from the stronger electron-donating character of triphenylamine than the *N*-phenylcarbazole moiety [31]. These results designate that the type, especially the electronic nature of substituents, plays a substantial role in elongating the π conjugation of the entire molecule, especially in their photophysical properties.

## 3. Materials and Methods

Procedure for the synthesis of **A**–**C**: In a 100 mL round-bottom flask, **3** (0.360 mmol), appropriate azide (0.890 mmol), ethanol (50 mL) and water (50 mL) were placed. The mixture was saturated with argon, and then CuSO_4_ 5H_2_O (0.220 g, 0.890 mmol), sodium ascorbate (0.200 g, 0.890 mmol), KF (0.055 g, 0.890 mmol) and pyridine (0.7 mL) were added. The mixture was stirred at room temperature for 24 h. Then, dichloromethane (20 mL) and a 5% ammonia solution (10 mL) were added, and the mixture was stirred for 30 min. The mixture was extracted with water (25 mL) and dichloromethane (2 × 25 mL). The combined organic layers were dried with anhydrous MgSO_4_, and the volatile fractions were evaporated. The crude product was purified by column chromatography (silica gel; CH_2_Cl_2_, ethyl acetate).

**A** was obtained as a yellow-orange solid (0.188 g, 62%) ^1^H NMR (400 MHz, CDCl_3_) δ 8.71 (d, *J* = 9.5 Hz, 2H), 8.64 (d, *J* = 5.6 Hz, 2H), 8.42 (d, *J* = 9.7 Hz, 2H), 8.40 (s, 2H), 7.91 (d, *J* = 10.9 Hz, 2H), 7.44–7.35 (m, 10H), 7.29 (s, 2H), 6.93 (d, *J* = 3.5 Hz, 2H), 5.69 (s, 4H), 3.75 (s, 4H), 1.08 (s, 18H). ^13^C NMR (101 MHz, CDCl_3_) δ 166.1, 160.5, 150.7, 147.8, 134.7, 129.4, 129.1, 129.0, 128.9, 128.3, 126.1, 126.0, 125.8, 125.6, 123.3, 112.4, 109.3, 78.1, 54.5, 32.0, 26.7. HRMS (ESI): *m/z* calcd. for C_54_H_51_N_8_O_2_ [MH^+^] 843.4129; found 843.4112 (see Supplementary Materials).

**B** was obtained as an orange solid (0.140 g, 52%) ^1^H NMR (400 MHz, CDCl_3_) δ 8.40 (s, 2H), 8.32–8.25 (m, 2H), 8.21 (s, 2H), 8.19–8.14 (m, 4H), 8.02 (s, *J* = 7.6 Hz, 2H), 7.72 (s, 2H), 4.42 (dt, *J* = 14.3, 7.2 Hz, 4H), 2.03–1.86 (m, 8H), 1.64 (s, 4H), 1.42–1.18 (m, 18H), 0.87 (t, *J* = 5.5 Hz, 8H). ^13^C NMR (101 MHz, CDCl_3_) δ 140.5, 139.3, 134.9, 132.3, 131.1, 129.7, 128.8, 126.3, 125.7, 125.2, 124.2, 123.3, 120.4, 119.2, 114.8, 107.2, 50.6, 32.0, 30.5, 30.4, 29.5, 29.5, 29.4, 29.2, 29.1, 26.7, 26.6, 22.8, 14.2. HRMS (ESI): *m*/*z* calcd. for C_46_H_57_N_10_ [MH^+^] 749.4762; found 749.4758.

**C** was obtained as a pale-yellow solid (0.101 g, 40%) ^1^H NMR (400 MHz, CDCl_3_) δ 8.61–8.50 (m, 4H), 8.43 (s, 2H), 7.94 (d, *J* = 19.4 Hz, 4H), 7.39 (s, 10H), 5.67 (s, 4H), 4.47 (t, *J* = 7.2 Hz, 4H), 2.08–1.96 (m, 4H), 1.39 (s, 8H), 1.35–1.17 (m, 18H), 0.86 (t, *J* = 6.1 Hz, 8H). ^13^C NMR (101 MHz, CDCl_3_) δ 146.9, 146.4, 134.8, 129.4, 129.0, 128.5, 128.4, 128.3, 125.8, 125.7, 125.6, 125.6, 125.5, 123.4, 123.3, 54.5, 50.7, 32.0, 30.5, 29.6, 29.54, 29.38, 29.2, 26.8, 22.8, 14.2. HRMS (ESI): *m*/*z* calcd. for C_58_H_67_N_12_ [MH^+^] 931.5606; found 931.5590.

## 4. Conclusions

The impact of the 1,2,3-triazole motif on the photophysical behavior of non-K tetrasubstituted pyrene derivatives with two kinds of substituents (4-(2,2-dimethylpropyloxy)pyridine, 1-decyl-1,2,3-triazole, 1-benzyl-1,2,3-triazole, and pyrazole) with a substitution pattern providing the long axial symmetry was presented in this paper based on the experimental investigations supported by theoretical calculations. All studied molecules are thermally stable; the highest temperatures corresponding to 5% and 10% weight loss during heating were observed for molecule **C**, at 354 °C and 383 °C, respectively. The least thermally stable was molecule **B**, with temperatures of 5% and 10% weight loss of 288 °C and 338 °C, respectively. These temperatures, compared to the analogs with the same four substituents at the positions 1, 3, 6 and 8 of pyrene, are mainly between the corresponding values. Compounds **A** and **C** with the same units of 1-benzyl-1,2,3-triazoles at the 1,8 positions have the absorption maxima at the same wavelength, whereas the low energetic band S_0_→S_1_ of compound **B** is shifted to a shorter wavelength of about 30 nm. The emission spectra of the compounds display a bright blue emission at 436 nm for **A**, 415 nm for **B** and 436 nm for **C**. The emission maximum for **A** and **C** was at the same wavelength (*λ*_em_ = 436 nm), revealing a bathochromic shift of 20 nm compared to compound **B**. All pyrene derivatives **A**–**C** are symmetrical, but in the case of **A** and **C**, the symmetry is strongly distorted by the arrangement of a decyl chain and benzyl at the triazole units in **C** and benzyl at triazole and neopentyloxy at pyridyl substituents in **A** and benzyl at the triazole unit, causing a bathochromic effect in relation to compound **B** with distortion symmetry only by the decyl alkyl chain in the triazole units at the 1,8 positions. All compounds exhibit high fluorescence quantum yields in the range of 71–81%. The quantum yield for compounds **A** and **C** is very similar, pointing out the exact character of the excited state and higher about 10% than that of compound **B**. Markedly, the fluorescence quantum yield depends on the type of substituents connected with the pyrene molecule. The difference in the quantum yield among **A**–**C** pyrene derivatives is reflected on the radiative process rate constant k_r_. The results further indicate that the **A**–**C** should exhibit a weak or no solvatochromic effect, even stressing the soft negative solvatochromic effect. The presence of the triazole units in the presented compounds indicates their role and influence on the photophysical properties of the 1,3,6,8-tetrasubstituted pyrene derivatives. The photophysical properties of the tetrasubstituted pyrene derivatives, having triazole units at the 1,8 positions, and other identical substituents at the 3,6 positions, show the dominance of the triazole units in the pyrene framework; the dominance is even higher in the case of the substitution of the 1,3,6,8 positions by triazoles, but containing two various alkyls. These results show that the type, especially the electronic nature of the substituents, plays a substantial role in elongating the π conjugation of the entire molecule, with significant changes, especially in their photophysical properties, in the efficient fluorophores that can be achieved based on the 1,3-dipolar cycloaddition by simply changing the substituted alkyl chains of the triazole groups.

## Figures and Tables

**Table 1:** Molecular orbitals calculated at the B3LYP/6-31G(d, p) level and their energies in eV for molecules **A**–**C**.

	HOMO-2	HOMO-1	HOMO	LUMO	LUMO+1	LUMO+2
A	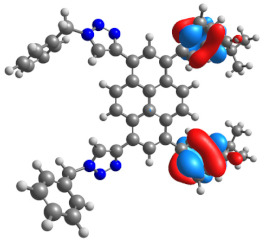	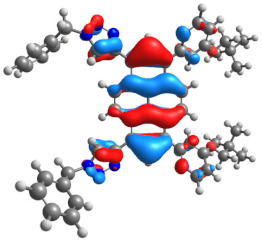	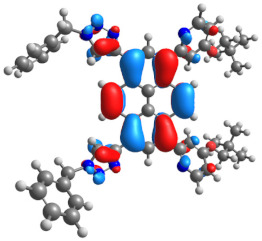	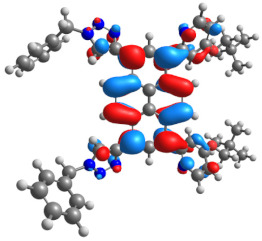	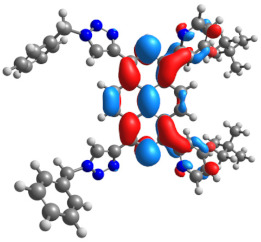	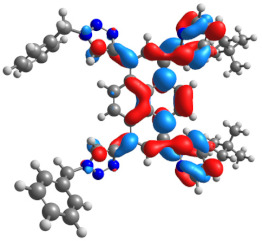
	−6.69	−6.15	−5.21	−2.01	−0.89	−0.58
B	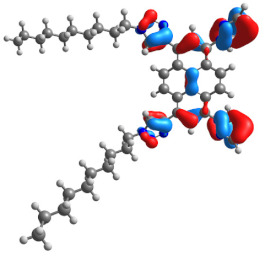	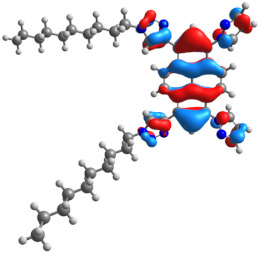	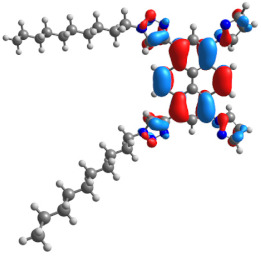	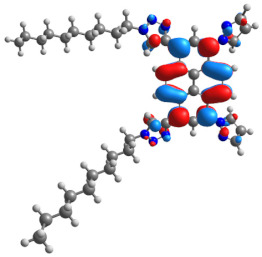	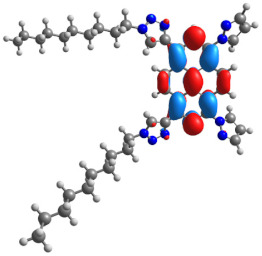	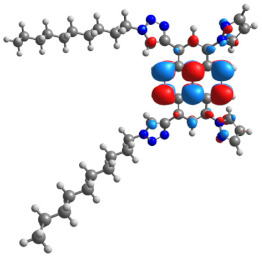
	−6.75	−6.37	−5.38	−2.13	−1.12	−0.59
C	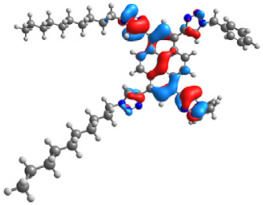	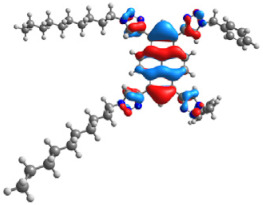	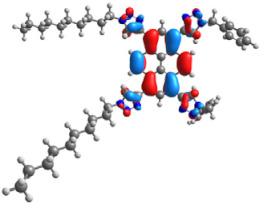	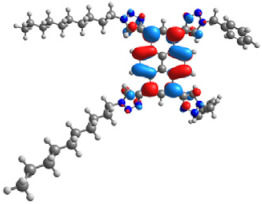	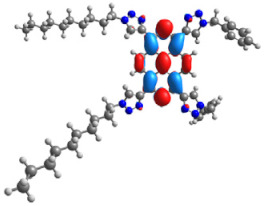	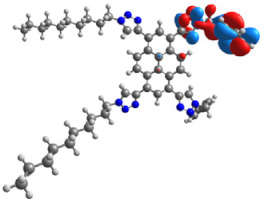
	−6.69	−6.18	−5.19	−2.02	−0.91	−0.49

**Table 2:** Chemical hardness (η), chemical potential (μ) and global electrophilicity indexes (ω) of molecules **A**–**C**.

	E_HOMO_ [eV]	E_LUMO_ [eV]	ΔE [eV]	η	μ	ω
**A**	−5.21	−2.01	3.20	1.60	−3.61	4.07
**B**	−5.38	−2.13	3.25	1.63	−3.76	4.34
**C**	−5.19	−2.02	3.17	1.59	−3.61	4.10

**Table 3:** Thermal properties of molecules **A**–**C**.

	T_5%_ [°C]	T_10%_ [°C]	T_max_ [°C]	Char Residue at 900 °C [%]
**A**	302	367	158; 247; 413	48
**B**	288	338	154; 408; 484; 700	6
**C**	354	383	283; 412; 696	3

T_5%_ and T_10%_: the temperature of 5% and 10% weight loss, respectively. T_max_: the maximum decomposition rate from the DTG thermograms.

**Table 4:** Photophysical and optical data recorded in the chloroform solution (*c* = 10^−5^ mol/L) for molecules **A**–**C**.

	λ_max_ [nm]	PL *λ*_em_ [nm]/ Stokes Shift [cm^−1^]	Φ [%]	τ [ns] (Weight%)	χ^2^	k_r_·10^8^ [s^−1^] ^[a]^	k_nr_·10^8^ [s^−1^] ^[a]^	E_g opt_ [eV] ^[b]^
**A**	258, 301, 392	436/2574	79	1.67 [0.05 (18.19) 2.03 (81.81)]	1.105	4.73	1.26	2.84
**B**	266, 279, 360	415/3681	71	2.83 [1.42 (27.19) 3.35 (72.81)]	1.159	2.51	1.02	2.99
**C**	257, 298, 391	436/2640	81	1.74 [0.02 (14.57) 2.03 (85.43)]	0.965	4.66	1.09	2.84

[a] Radiative (k_r_) and non-radiative (k_nr_) decay rates assuming that emission excited states are produced with unit efficiency and are estimated using the following equations: k_r_ = Φ_em_/τ; k_nr_ = (1-Φem)/τ; [b] E_g opt_ = 1241/λ_em_.

**Table 5:** The calculated absorption spectra by the TD-DFT method (6-31G(d,p)/CAM-B3LYP) with oscillator strengths for **A**–**C**.

	Calculated Wavelengths [nm] (Oscillator Strengths)	Transitions (Contribution)
**A**	368.22 (1.0109)	HOMO→LUMO (96%)
	269.08 (1.4097)	HOMO→L+1 (53%), H-1→LUMO (43%)
	231.37 (0.4513)	H-1→L+1 (34%)
	230.06 (0.3183)	H-1→L+1 (33%), H-12→LUMO (17%)
	220.93 (0.2108)	H-11→LUMO (42%), HOMO→L+6 (14%)
**B**	361.09 (0.9955)	HOMO→LUMO (96%)
	267.16 (1.2264)	H-1→LUMO (55%), HOMO→L+1 (43%)
	229.57 (0.8705)	H-1→L+1 (72%)
**C**	370.73 (0.9852)	HOMO→LUMO (96%)
	269.12 (1.3156)	H-1→LUMO (49%), HOMO→L+1 (49%)
	229.32 (0.8205)	H-1→L+1 (83%)
	222.43 (0.1078)	H-9→LUMO (86%)

**Table 6:** The calculated by TD-DFT (6-31G(d,p)/CAM-B3LYP) wavelengths of emission with oscillator strengths for **A**–**C**.

	Calculated Wavelengths [nm] (Oscillator Strengths)	Transitions (Contribution)
**A**	473.57 (1.1810)	HOMO→LUMO (98%)
**B**	454.54 (1.2025)	HOMO→LUMO (98%)
**C**	470.64 (1.1534)	HOMO→LUMO (98%)

**Table 7:** Calculated dipole moment B3LYP/6-31G(d,p) for molecules **A**–**C**.

	Ground State (μ_g_) [D]	Excited State (μ_e_) [D]	Δμ [D]
**A**	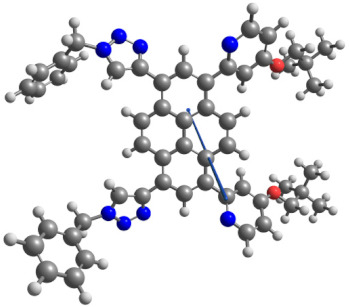	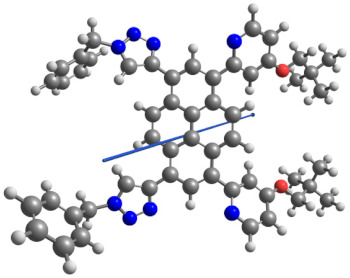	−0.89
	2.45	1.56	
**B**	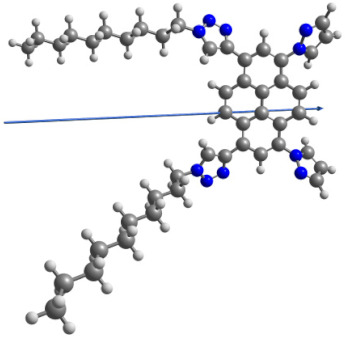	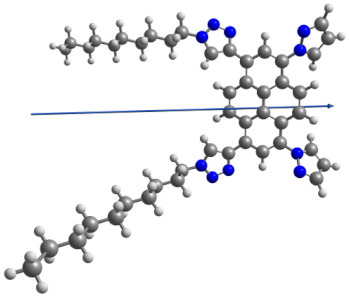	−0.30
	8.23	7.93	
**C**	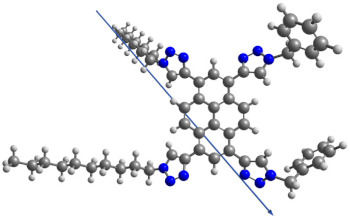	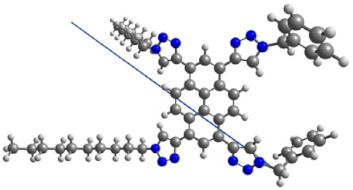	−0.22
	1.19	0.97	

**Table 8:** Photophysical and optical data recorded in the solution (CHCl_3_ or CH_2_Cl_2_) (*c* = 10^−5^ mol/L) of the 1,3,6,8-tetrasubstituted pyrene substituted by two kinds of substituents with the substitution pattern providing the long axial symmetry reported in the literature.

			λ_abs_ [nm]	PL *λ*_em_ [nm]/ Stokes Shift [cm^−1^]	Φ [%]	E_g opt_ [eV]	Ref.
**1L**	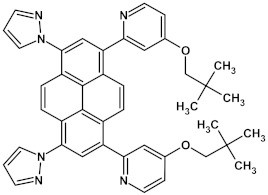	CHCl_3_	259, 282, 379	428/3021	89	2.90	[16]
**2L**	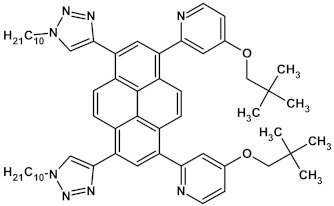	CH_2_Cl_2_	259, 285, 297, 390	438/2810	95	2.83	[17]
**3L**	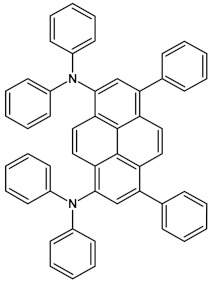	CH_2_Cl_2_	257, 299, 340, 436	479/2059	89	2.64	[30]
**4L**	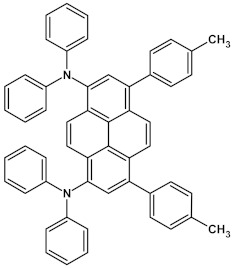	CH_2_Cl_2_	257, 299, 340, 436	476/1927	85	2.61	[30]
**5L**	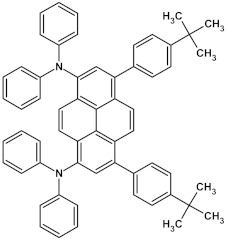	CH_2_Cl_2_	258, 300, 437	488/2349	67	2.50	[31]
**6L**	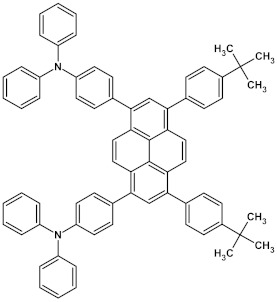	CH_2_Cl_2_	251, 301, 405	483/3944	88	2.78	[31]
**7L**	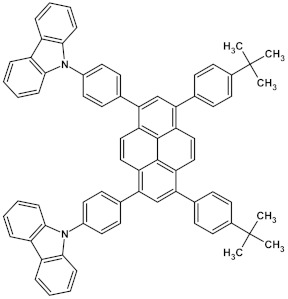	CH_2_Cl_2_	241, 294, 391	437/2692	95	2.96	[31]

## Data Availability

Not applicable.

## Supplementary Materials

The following supporting information can be downloaded at: https://www.mdpi.com/article/10.3390/molecules27134314/s1, Figure S1–S6: ^1^H, ^13^C NMR spectra of target compounds A-C, Figure S7–S11: HRMS spectra of target molecules A–C; Description of Materials, Instruments, and Cartesian coordinates of the DFT-optimized structures.
