# Supplementary material for: The Impact of a 1,2,3-Triazole Motif on the Photophysical Behavior of Non-K Tetrasubstituted Pyrene with a Substitution Pattern Providing the Long Axial Symmetry
Source: Molecules. 2022 Jul 5;27(13):4314. doi: 10.3390/molecules27134314 (PMC9268544; doi:10.3390/molecules27134314)
Supplement: Supplementary file 1 [file molecules-27-04314-s001.zip › molecules-1787955-supplementary.pdf]

The Impact of a 1,2,3-Triazole Motif on the Photophysical Behavior of Non-K Tetrasubstituted Pyrene with a Substitution Pattern Providing the Long Axial Symmetry

Dawid Zych [1,*] and Aneta Slodek [2]

[1]Faculty of Chemistry, University of Opole, Oleska 48, 45-052 Opole, Poland

[2]Institute of Chemistry, Faculty of Science and Technology, University of Silesia, Szkolna 9, 40-006 Katowice, Poland

*Correspondence: dawidzych92@gmail.com, dawid.zych@uni.opole.pl; Tel.:+48 77 452-71-17

1. Materials

All chemicals and starting materials were commercially available and were used without further purification. Solvents were distilled as per the standard methods and purged with nitrogen before use. All reactions were carried out under argon atmosphere unless otherwise indicated. Column chromatography was carried out on Merck silica gel. Thin-layer chromatography (TLC) was performed on silica gel (Merck TLCSilicaGel60).

2. Instruments

NMR spectra were measured in deuterated chloroform using Bruker Avance 400 MHz (^{1}H and ^{13}C NMR).

High-resolution mass spectrometry (HRMS) measurements were conducted using Mass Spectrometer QTOF (Impact HD, Bruker).

Thermogravimetric analysis (TGA) was carried out using Pyris 1 TGA Perkin-Elmer.

UV-Vis spectra were measured using Perkin–Elmer Lambda Bio 40 UV-Vis spectrophotometer at room temperature with a conventional 1.0 cm quartz cell.

The emission and excitation spectra were measured by using the spectrophotometer Hitachi F-7000.

The quantum fluorescence yields were determined by the absolute method at room temperature, using the integrating sphere with solvent as a blank (FLS-980 spectrophotometer). The time-resolved measurement has been prepared at optically diluted solutions at room temperature using the time-correlated single-photon counting methods on the FLS-980 spectrophotometer. Excitation wavelengths were obtained using the picosecond pulsed diode EPLED–375 nm and nm with 50 ns pulse period as light sources. PMT (Hamamatsu, R928P)

in cooled housing was used as a detector. The system was levelled at emission wavelengths. In addition, an instrument response function was obtained for the analysis of fluorescence decay. The IRF contains information about the time response of the overall optical and electronic system. The IRF was designated using LUDOX solution as a standard at 375 nm.

3. NMR spectra

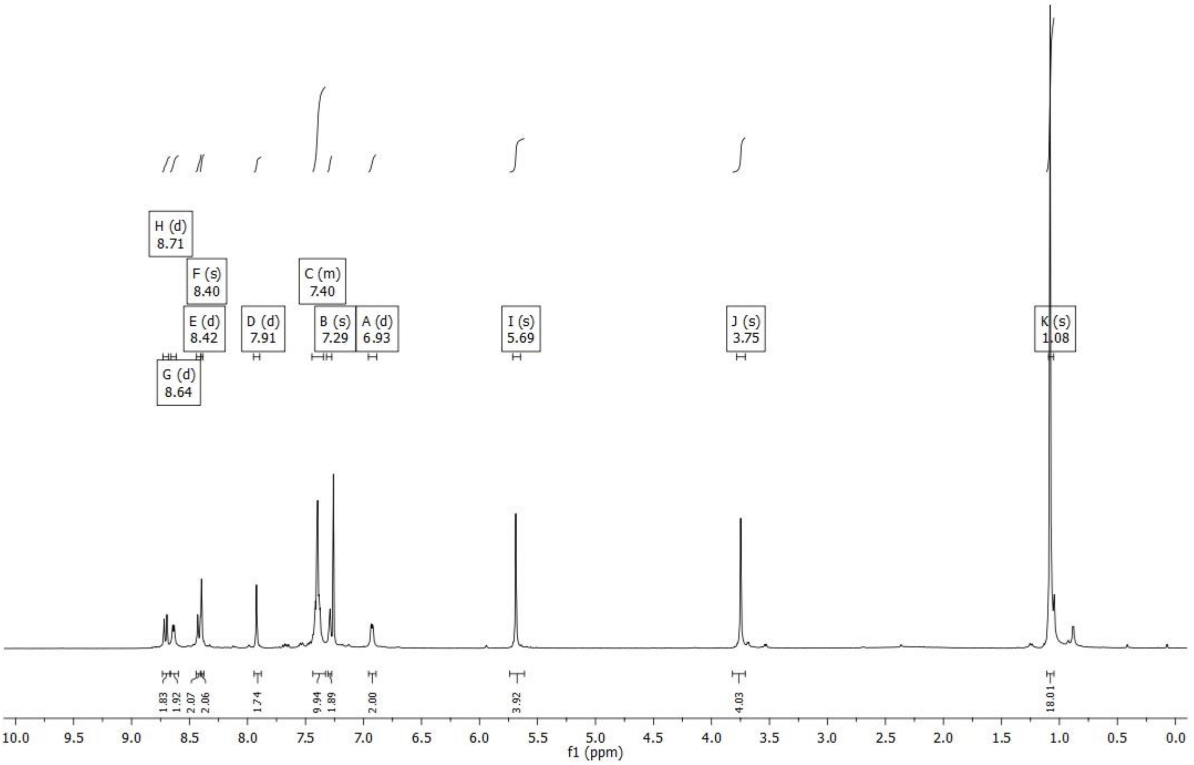

Figure S1. ^{1}H NMR spectra of **A**.

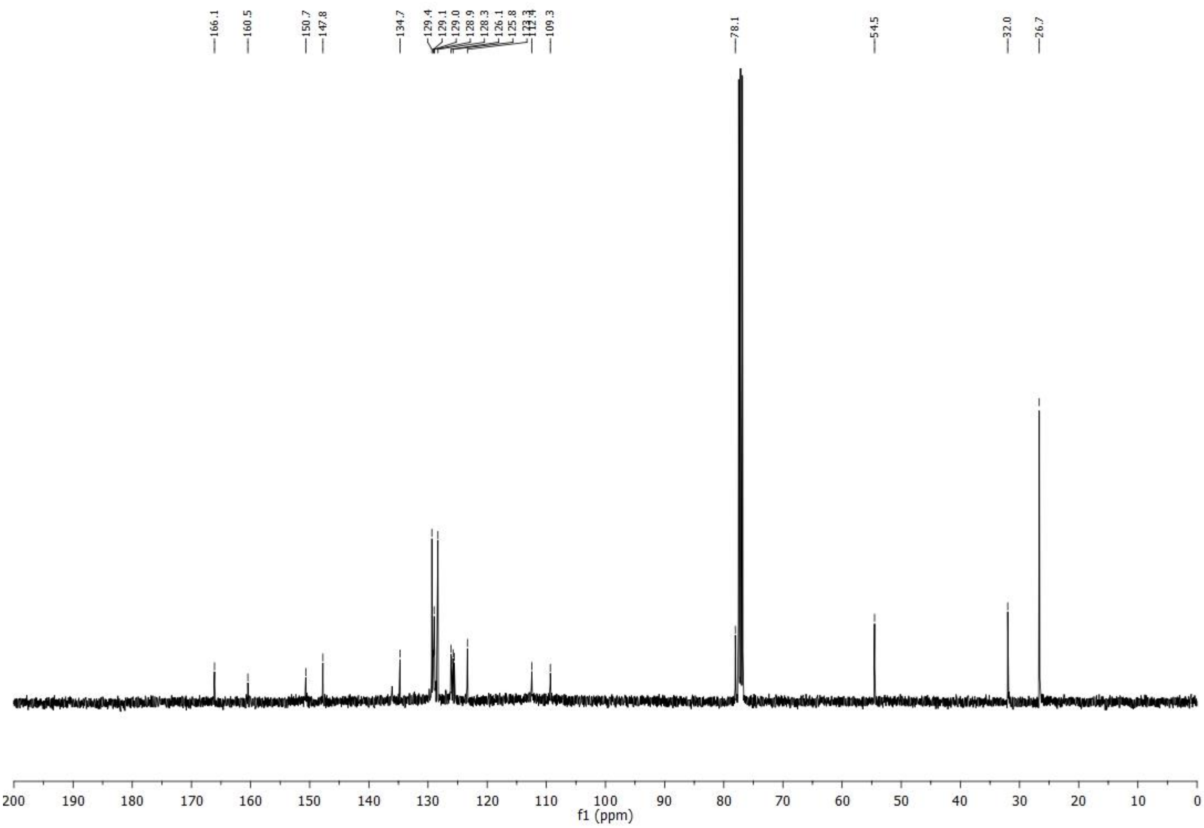

Figure S2. ^{13}C NMR spectra of **A**.

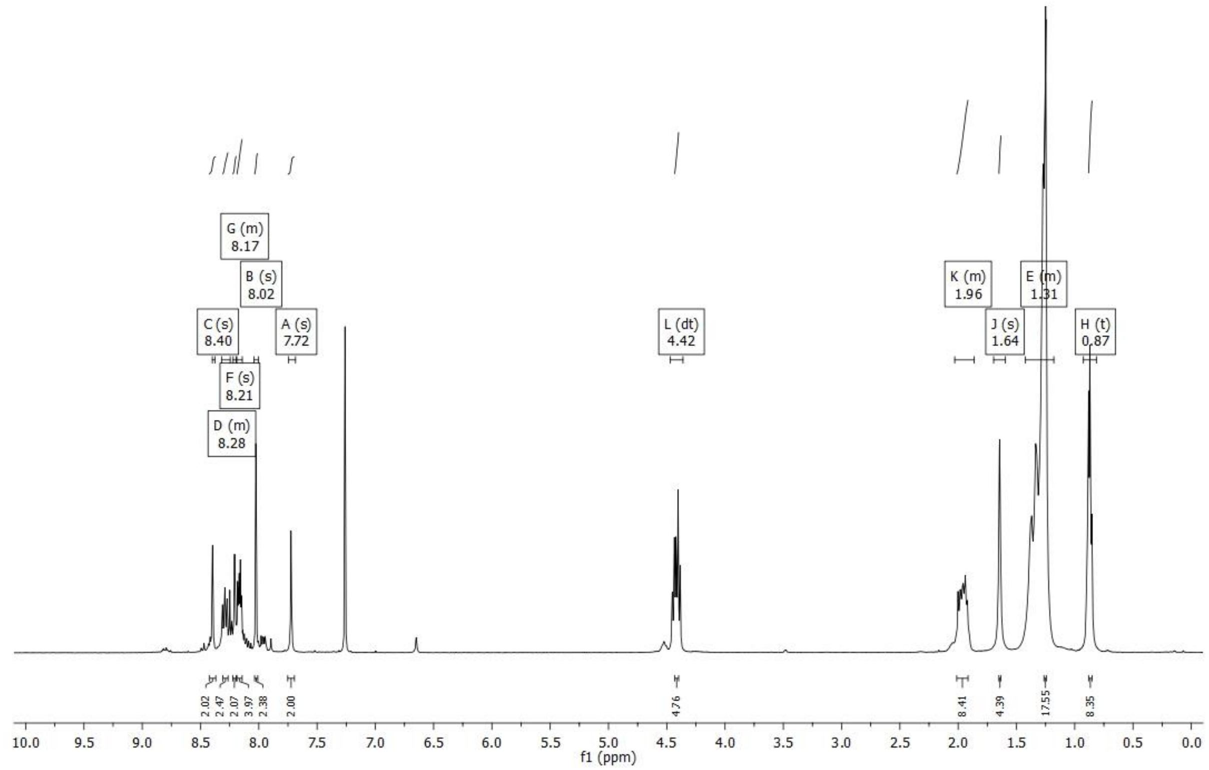

Figure S3. ^{1}H NMR spectra of **B**.

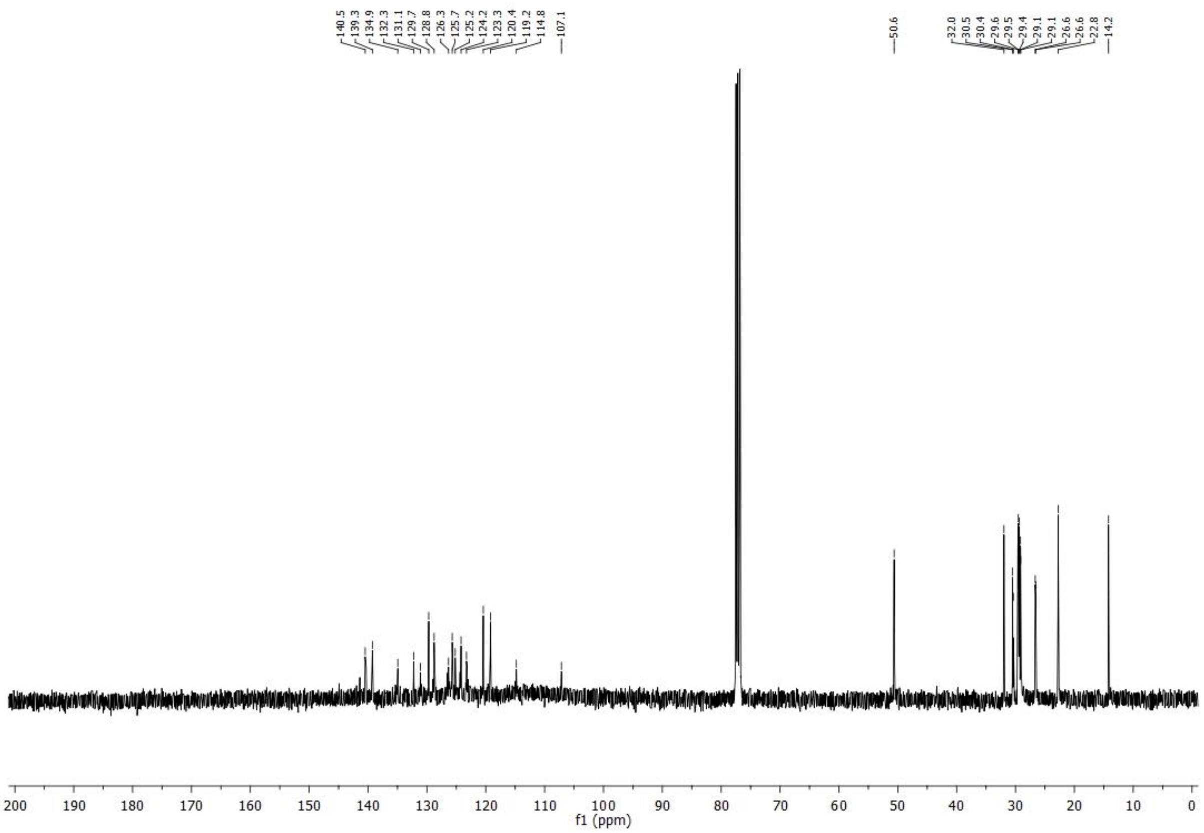

Figure S4. ^{13}C NMR spectra of **B**.

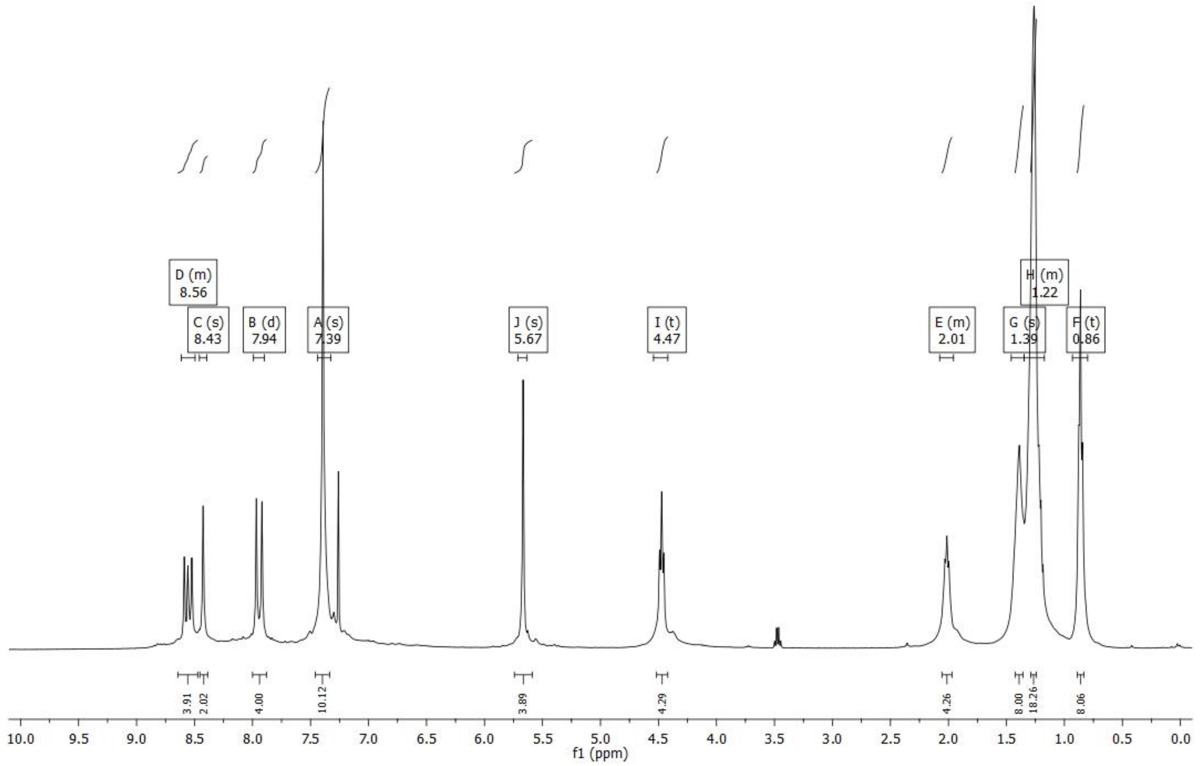

Figure S5. ^{1}H NMR spectra of **C**.

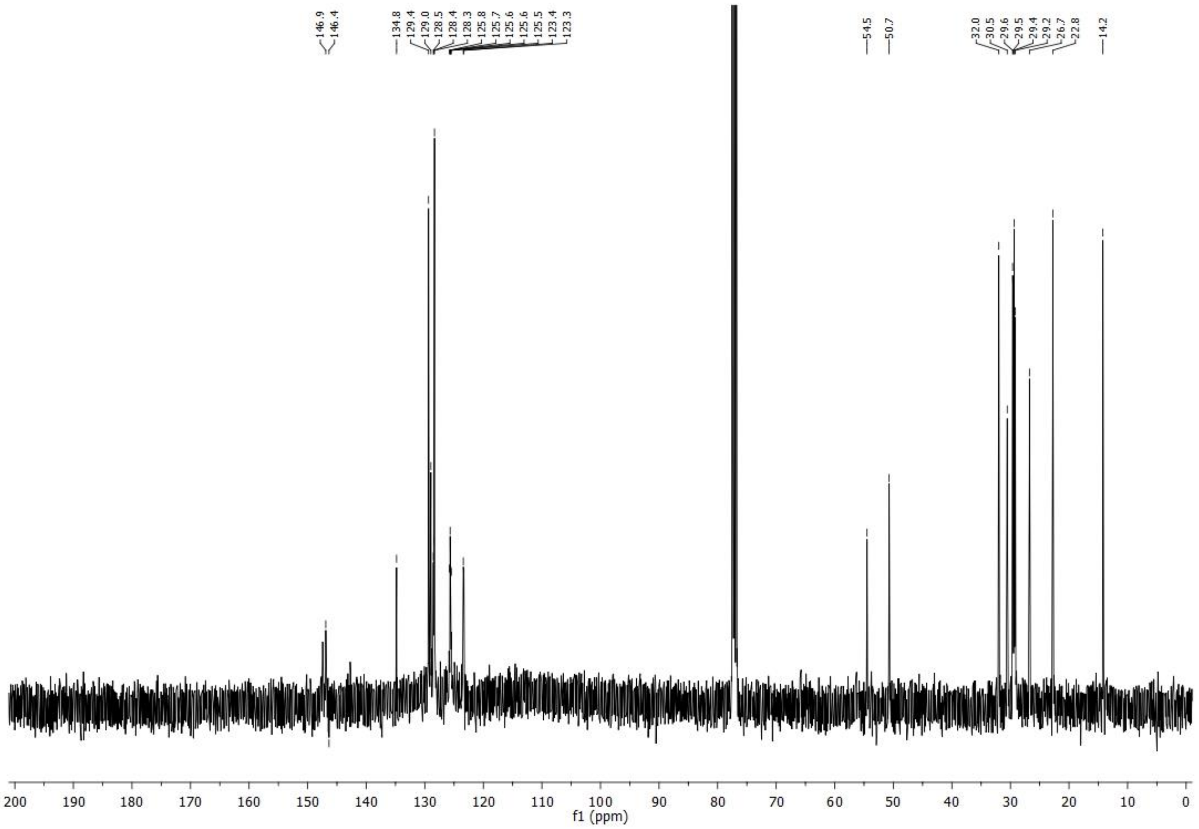

Figure S6. ^{13}C NMR spectra of **C**.

4. MS spectra

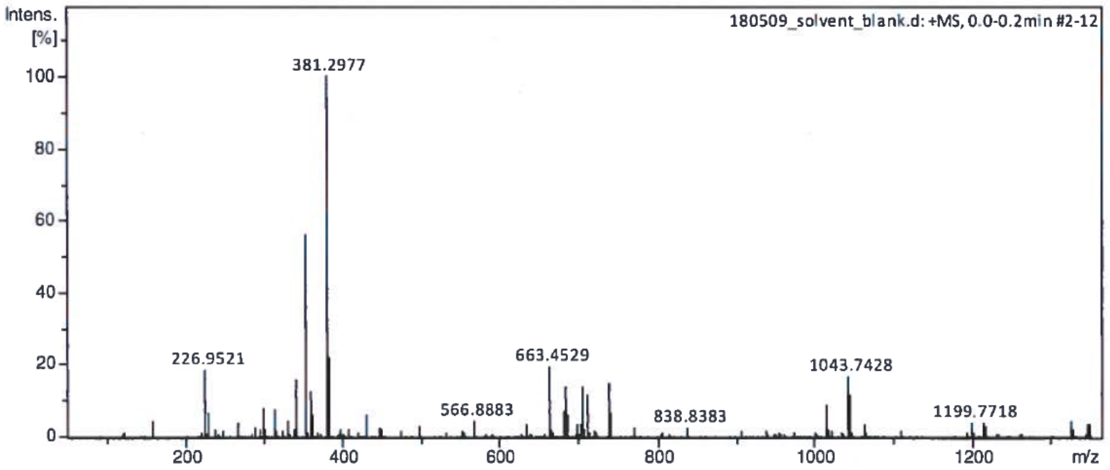

Figure S7. MS spectrum of solvents in positive mode ESI-MS.

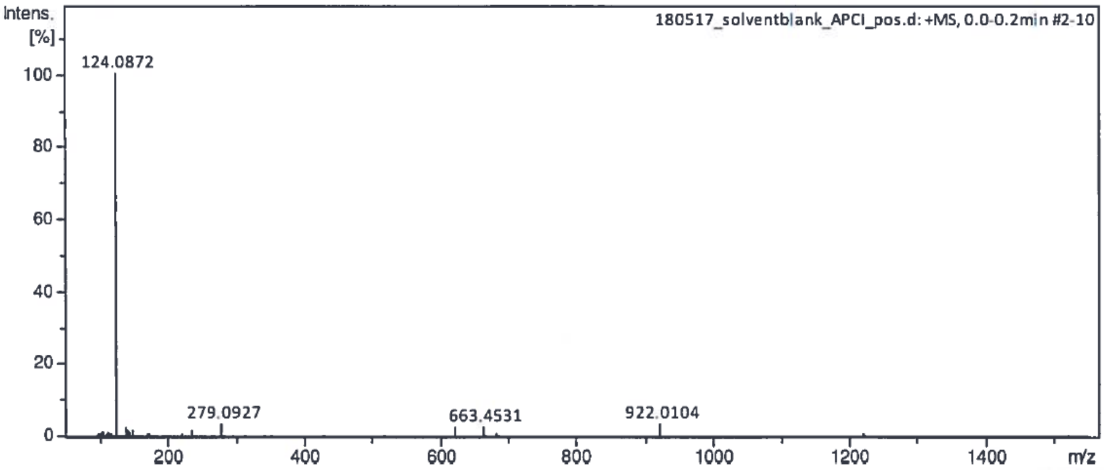

Figure S8. MS spectrum of solvents in positive mode APCI-MS.

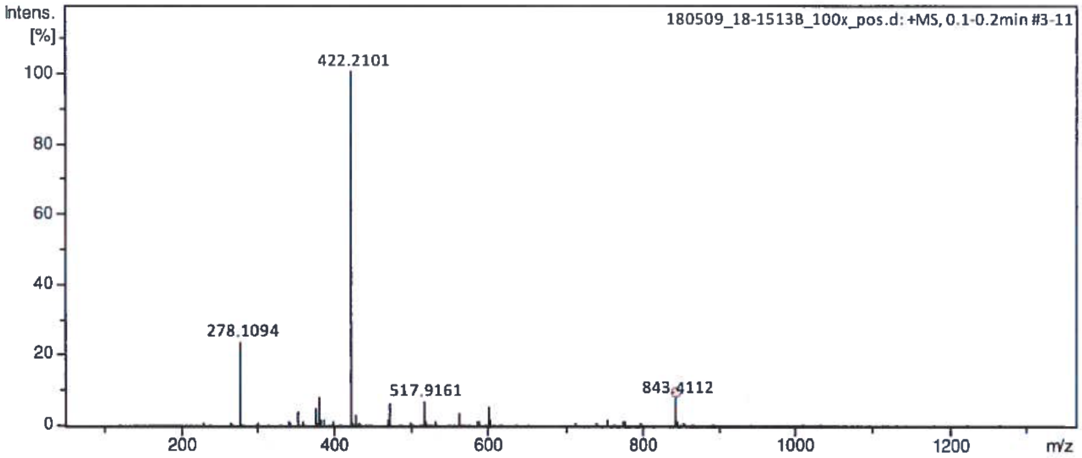

The isotopic pattern of peak 843 m/z (experimental at the top, theoretical at the bottom)

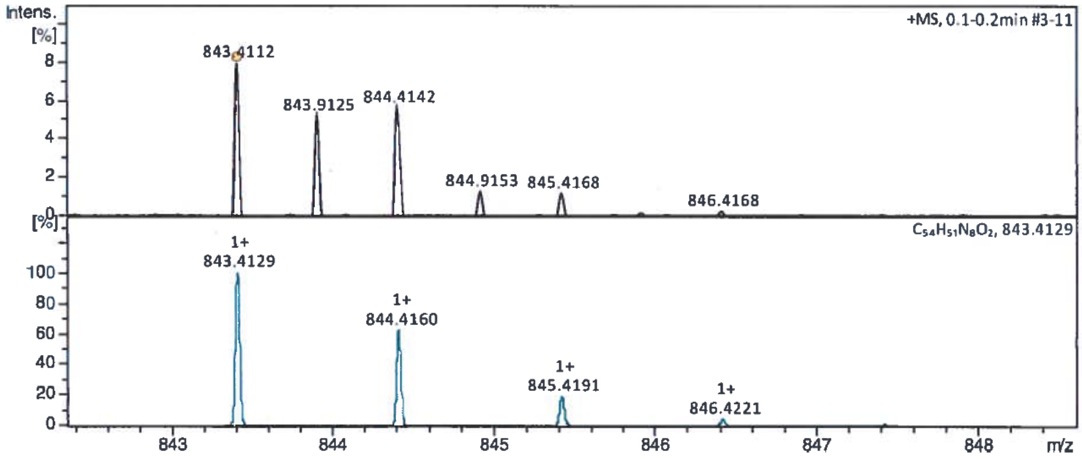

Figure S9. MS spectrum of **A**.

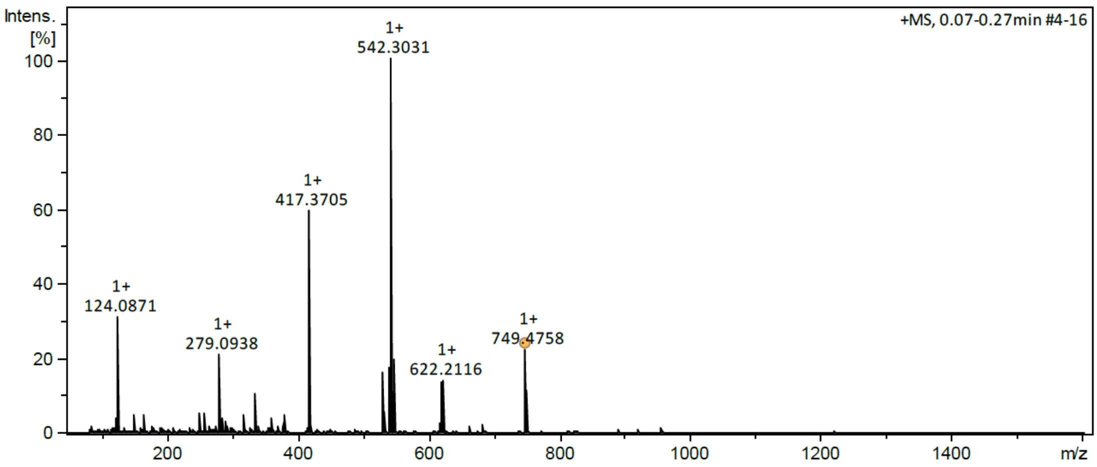

The isotopic pattern of peak 749 m/z (experimental at the top, theoretical at the bottom)

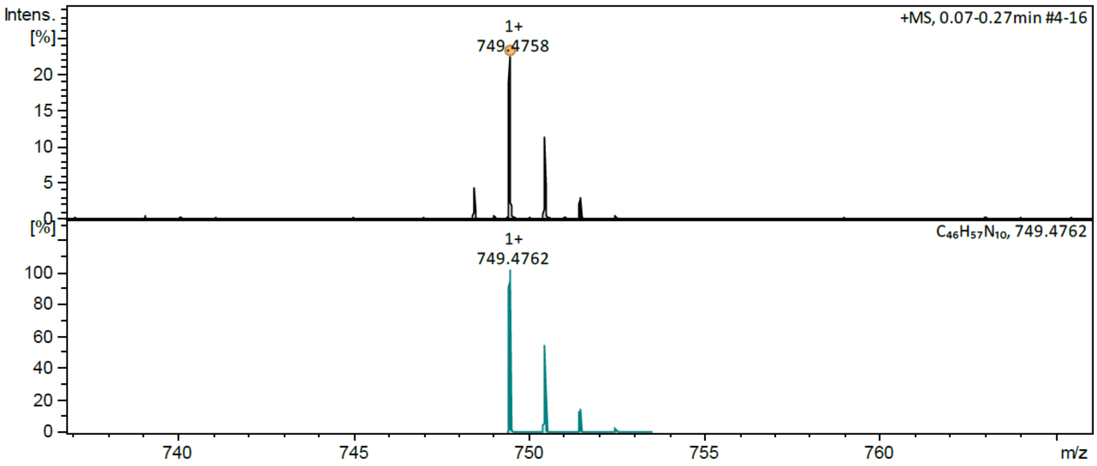

Figure S10. MS spectrum of **B**.

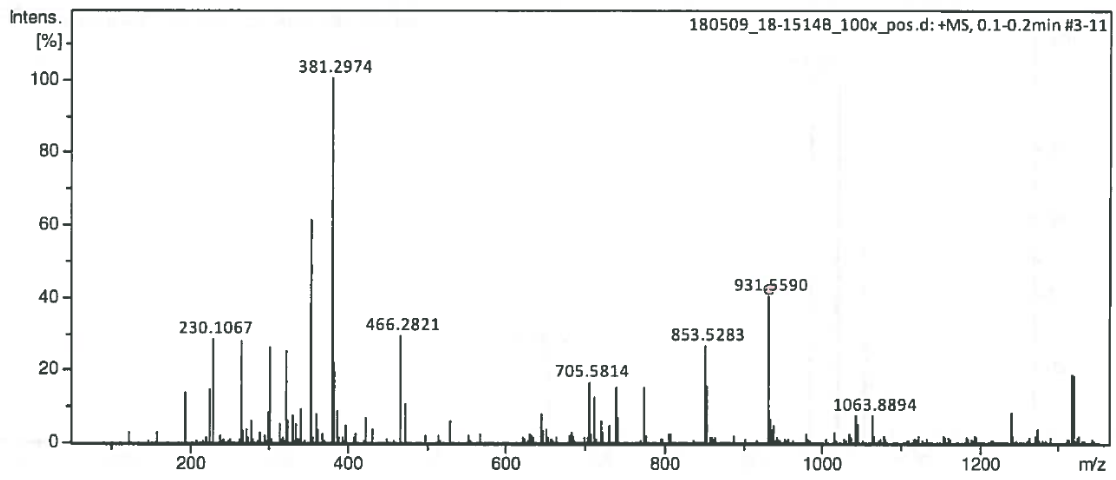

The isotopic pattern of peak 931 m/z (experimental at the top, theoretical at the bottom)

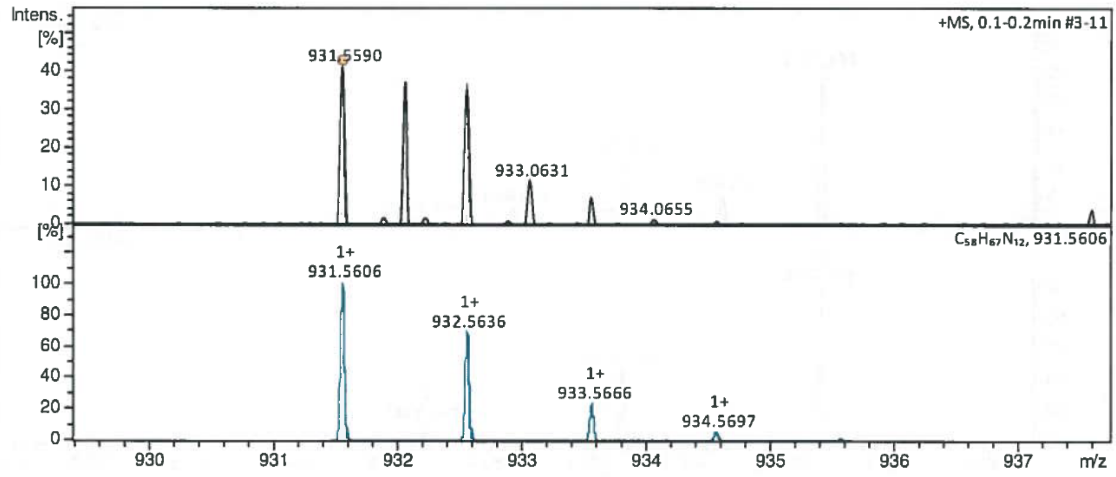

Figure S11. MS spectrum of **C**.

5. Cartesian coordinates

Cartesian coordinates of DFT-optimized structure of **A** by B3LYP/6-31G(d,p)/$CHCl_3$
Charge = 0; multiplicity=1

C	-0.671430000	3.272532000	0.189641000
C	0.572470000	2.766706000	-0.188766000
C	0.717612000	1.372321000	-0.406787000
C	-0.388049000	0.501898000	-0.132090000
C	-1.633965000	1.045935000	0.320596000
C	-1.777044000	2.453764000	0.431054000
C	1.908700000	0.793803000	-0.957281000
C	2.041995000	-0.549886000	-1.126198000
C	0.998526000	-1.464481000	-0.764230000
C	-0.247447000	-0.918337000	-0.311583000
C	-2.674504000	0.132289000	0.688200000
C	-2.541985000	-1.211552000	0.517653000
C	-1.353547000	-1.787035000	-0.038324000
C	1.130752000	-2.870513000	-0.899738000
C	0.017166000	-3.684335000	-0.689474000
C	-1.219359000	-3.178735000	-0.282173000
C	-3.041451000	3.113963000	0.796924000
N	-3.052100000	4.271776000	1.535178000
N	-4.279676000	4.693112000	1.668397000
N	-5.082977000	3.822292000	1.018866000
C	-4.355080000	2.827890000	0.458578000
H	-0.781765000	4.344523000	0.300590000
H	2.712137000	1.448976000	-1.270686000
H	2.949256000	-0.940328000	-1.570609000
H	-3.574914000	0.520907000	1.148024000
H	-3.339359000	-1.867998000	0.844392000
H	0.117647000	-4.751152000	-0.849635000
H	-4.803552000	2.051697000	-0.139691000
C	-2.325634000	-4.137300000	-0.122829000
C	-3.672019000	-4.036692000	-0.436401000
N	-4.185507000	-5.258109000	-0.160030000
N	-3.224122000	-6.085321000	0.305586000
N	-2.103757000	-5.416481000	0.324208000
H	-4.270086000	-3.238542000	-0.845016000
C	-6.528514000	4.053647000	0.966063000
C	-7.019163000	4.455600000	-0.411097000
C	-8.042016000	3.733291000	-1.033197000
C	-8.516234000	4.116392000	-2.290402000
C	-7.964277000	5.222262000	-2.936953000

C	-6.938874000	5.946565000	-2.321476000
C	-6.470549000	5.567483000	-1.064707000
C	-5.569427000	-5.724487000	-0.273432000
C	-6.497862000	-5.085138000	0.740572000
C	-6.227334000	-5.184921000	2.112328000
C	-7.091821000	-4.612415000	3.043698000
C	-8.239360000	-3.938169000	2.615202000
C	-8.515458000	-3.837509000	1.251620000
C	-7.645053000	-4.406350000	0.318181000
C	1.684882000	3.747713000	-0.351217000
N	1.376238000	4.893607000	-1.001064000
C	2.339741000	5.810548000	-1.114818000
C	3.635430000	5.676607000	-0.614384000
C	3.943938000	4.496759000	0.072737000
C	2.944868000	3.521989000	0.206866000
C	2.408267000	-3.546592000	-1.269976000
C	3.611209000	-3.229943000	-0.635544000
C	4.777202000	-3.929625000	-0.977916000
C	4.689720000	-4.935460000	-1.947529000
C	3.435193000	-5.185620000	-2.505001000
N	2.314584000	-4.529742000	-2.194707000
O	5.139081000	4.211902000	0.637478000
C	6.198851000	5.177710000	0.550846000
O	5.904862000	-3.572286000	-0.322858000
C	7.440734000	4.628839000	1.268648000
C	8.545599000	5.695537000	1.146404000
C	7.902609000	3.321816000	0.597606000
C	7.121567000	4.377388000	2.754123000
C	7.128739000	-4.266645000	-0.611573000
H	-6.714855000	4.841049000	1.700736000
H	-7.036130000	3.147783000	1.306981000
H	-8.471781000	2.869260000	-0.532775000
H	-9.311487000	3.547907000	-2.763266000
H	-8.328989000	5.519628000	-3.915568000
H	-6.506053000	6.808427000	-2.820468000
H	-5.673540000	6.131216000	-0.587635000
H	-5.515031000	-6.807425000	-0.137408000
H	-5.916131000	-5.529494000	-1.291531000
H	-5.336208000	-5.709138000	2.446694000
H	-6.872889000	-4.694632000	4.104105000
H	-8.913223000	-3.494740000	3.342148000
H	-9.404336000	-3.314598000	0.911444000
H	-7.861718000	-4.324268000	-0.743828000
H	2.069695000	6.722836000	-1.644234000

H	4.355499000	6.472043000	-0.755454000
H	3.163452000	2.618089000	0.762845000
H	3.660998000	-2.469368000	0.134532000
H	5.547118000	-5.516454000	-2.261229000
H	3.336973000	-5.969850000	-3.253874000
H	5.872685000	6.117030000	1.016806000
H	6.424880000	5.374937000	-0.505553000
H	8.793090000	5.898728000	0.098395000
H	9.458849000	5.354010000	1.644099000
H	8.244753000	6.640026000	1.613473000
H	8.147790000	3.485002000	-0.458101000
H	7.125145000	2.554865000	0.650086000
H	8.798457000	2.933473000	1.093351000
H	6.805937000	5.301356000	3.251886000
H	8.006714000	4.000505000	3.277165000
H	6.320502000	3.641872000	2.866651000
C	8.252266000	-3.693638000	0.264819000
C	7.912260000	-3.893186000	1.753578000
C	8.444893000	-2.196049000	-0.038321000
C	9.539349000	-4.465443000	-0.083502000
H	7.373971000	-4.143241000	-1.674840000
H	6.995047000	-5.337399000	-0.408457000
H	8.716434000	-3.501674000	2.385343000
H	7.786585000	-4.955781000	1.990694000
H	6.987025000	-3.375280000	2.020213000
H	7.535499000	-1.629294000	0.179008000
H	8.700990000	-2.036100000	-1.091934000
H	9.256863000	-1.784179000	0.570098000
H	9.431808000	-5.536974000	0.119061000
H	10.377079000	-4.095590000	0.516305000
H	9.806323000	-4.344678000	-1.139420000

Cartesian coordinates of DFT-optimized structure of **B** by B3LYP/6-31G(d,p)/$CHCl_3$
Charge = 0; multiplicity=1

C	3.126228000	-3.638340000	-1.540325000
C	4.438357000	-3.454433000	-1.122110000
C	4.849843000	-2.246686000	-0.516284000
C	3.903221000	-1.174444000	-0.439719000
C	2.564723000	-1.356669000	-0.917120000
C	2.171812000	-2.624287000	-1.422636000
C	6.147177000	-2.065927000	0.064720000
C	6.523273000	-0.873568000	0.601566000

C	5.638186000	0.253300000	0.610107000
C	4.299973000	0.083981000	0.128137000
C	1.689000000	-0.222579000	-0.906566000
C	2.064406000	0.969452000	-0.367020000
C	3.357587000	1.159055000	0.220171000
C	6.006482000	1.518712000	1.118373000
C	5.074022000	2.540670000	1.247195000
C	3.747941000	2.376626000	0.837930000
C	0.800218000	-2.938696000	-1.853851000
N	0.563951000	-3.810781000	-2.886781000
N	-0.722712000	-3.974823000	-3.032360000
N	-1.339967000	-3.218302000	-2.100305000
C	-0.431969000	-2.560872000	-1.342170000
H	2.839615000	-4.594160000	-1.960266000
H	6.830567000	-2.905603000	0.098889000
H	7.501570000	-0.778260000	1.056652000
H	0.717363000	-0.307015000	-1.377224000
H	1.384085000	1.810634000	-0.417927000
H	5.384682000	3.482024000	1.682341000
H	-0.719183000	-1.928001000	-0.518375000
C	2.828864000	3.503363000	1.064148000
C	1.493245000	3.534980000	1.435694000
N	1.207170000	4.848366000	1.593257000
N	2.292964000	5.606288000	1.331740000
N	3.272153000	4.801241000	1.020554000
H	0.770955000	2.759533000	1.633044000
N	5.348455000	-4.535511000	-1.297728000
N	4.979921000	-5.799434000	-0.947623000
C	6.001237000	-6.564687000	-1.326820000
C	7.032484000	-5.812929000	-1.934048000
C	6.574649000	-4.513183000	-1.904505000
N	7.340286000	1.779804000	1.543338000
C	8.499017000	1.593233000	0.839888000
C	9.524574000	2.090597000	1.614873000
C	8.885248000	2.574649000	2.778633000
N	7.566614000	2.395934000	2.737148000
H	5.963633000	-7.630934000	-1.146616000
H	7.967954000	-6.166415000	-2.340614000
H	6.998823000	-3.594157000	-2.278900000
H	8.492468000	1.152961000	-0.145378000
H	10.576386000	2.108403000	1.372543000
H	9.332484000	3.041462000	3.646299000
C	-0.070477000	5.480919000	1.923795000
C	-0.947844000	5.722167000	0.690537000

C	-2.269285000	6.410757000	1.051966000
C	-3.165696000	6.665337000	-0.166316000
C	-4.488216000	7.357103000	0.185773000
C	-5.386931000	7.612864000	-1.030359000
C	-6.709399000	8.305818000	-0.679985000
C	-7.609109000	8.561768000	-1.895146000
C	-8.931616000	9.255055000	-1.545387000
C	-9.824179000	9.506630000	-2.764469000
C	-2.797826000	-3.246504000	-1.974972000
C	-3.282698000	-4.247921000	-0.921033000
C	-4.812884000	-4.284469000	-0.828027000
C	-5.328086000	-5.282961000	0.216018000
C	-6.858190000	-5.328947000	0.308170000
C	-7.379082000	-6.328199000	1.348408000
C	-8.909364000	-6.376700000	1.437726000
C	-9.432774000	-7.376456000	2.475997000
C	-10.963128000	-7.425962000	2.564056000
C	-11.477816000	-8.427058000	3.602793000
H	0.175548000	6.423699000	2.417524000
H	-0.578219000	4.840494000	2.650532000
H	-1.149259000	4.762846000	0.197270000
H	-0.386742000	6.335424000	-0.024466000
H	-2.056547000	7.365316000	1.552570000
H	-2.813949000	5.796357000	1.782509000
H	-3.376885000	5.709944000	-0.667058000
H	-2.618102000	7.277231000	-0.896516000
H	-4.275196000	8.312045000	0.686749000
H	-5.034004000	6.745332000	0.918215000
H	-5.599647000	6.657643000	-1.531408000
H	-4.840248000	8.223821000	-1.762686000
H	-6.496178000	9.261076000	-0.179282000
H	-7.255603000	7.695089000	0.053183000
H	-7.823263000	7.606819000	-2.396478000
H	-7.063711000	9.172753000	-2.628760000
H	-8.717685000	10.209487000	-1.045286000
H	-9.476631000	8.644581000	-0.812451000
H	-9.319341000	10.143481000	-3.499930000
H	-10.758899000	10.002014000	-2.481860000
H	-10.083951000	8.567068000	-3.265636000
H	-3.180989000	-3.505570000	-2.964492000
H	-3.130672000	-2.232072000	-1.737597000
H	-2.855062000	-3.982900000	0.054095000
H	-2.896763000	-5.241602000	-1.177584000
H	-5.230498000	-4.539854000	-1.811673000

H	-5.188783000	-3.280153000	-0.587115000
H	-4.911329000	-5.026580000	1.200141000
H	-4.947699000	-6.285523000	-0.023975000
H	-7.272630000	-5.582976000	-0.677769000
H	-7.237679000	-4.325214000	0.547294000
H	-6.965791000	-6.073718000	2.334843000
H	-6.997765000	-7.331244000	1.109833000
H	-9.321792000	-6.630281000	0.450636000
H	-9.290592000	-5.373395000	1.676336000
H	-9.021813000	-7.123290000	3.463959000
H	-9.051820000	-8.380083000	2.238188000
H	-11.373516000	-7.679282000	1.577007000
H	-11.343862000	-6.423301000	2.802068000
H	-11.142283000	-9.444639000	3.371957000
H	-12.572076000	-8.438579000	3.640983000
H	-11.112505000	-8.179566000	4.606129000

Cartesian coordinates of DFT-optimized structure of **C** by B3LYP/6-31G(d,p)/$CHCl_3$
Charge = 0; multiplicity=1

C	-1.673476000	2.854993000	-0.867477000
C	-2.931708000	2.393435000	-0.475462000
C	-3.073531000	1.051675000	-0.034589000
C	-1.944296000	0.171716000	-0.097361000
C	-0.677107000	0.661090000	-0.553637000
C	-0.544551000	2.033227000	-0.892241000
C	-4.292486000	0.546241000	0.522631000
C	-4.424550000	-0.756413000	0.895075000
C	-3.351141000	-1.693934000	0.751011000
C	-2.083278000	-1.204351000	0.297286000
C	0.395743000	-0.277198000	-0.697208000
C	0.264600000	-1.579285000	-0.322151000
C	-0.954084000	-2.084586000	0.236061000
C	-3.483828000	-3.066726000	1.086628000
C	-2.354934000	-3.888102000	1.066746000
C	-1.095266000	-3.426034000	0.678703000
C	0.732158000	2.650625000	-1.288967000
N	0.778125000	3.651797000	-2.226587000
N	2.006872000	4.075437000	-2.351823000
N	2.774564000	3.361778000	-1.501537000
C	2.023038000	2.467937000	-0.816797000
C	4.200052000	3.663375000	-1.368285000
C	4.490610000	4.666557000	-0.246592000

C	5.984067000	5.000176000	-0.149012000
C	6.304291000	6.002356000	0.967006000
C	7.795582000	6.346234000	1.064913000
C	8.120742000	7.347203000	2.180378000
C	9.611596000	7.693851000	2.276585000
C	9.938882000	8.693883000	3.392068000
C	11.429522000	9.041840000	3.487381000
C	11.747964000	10.040600000	4.604144000
H	-1.568871000	3.891387000	-1.165341000
H	-5.117167000	1.229288000	0.685605000
H	-5.351828000	-1.086175000	1.347622000
H	1.322012000	0.052165000	-1.152029000
H	1.088946000	-2.262817000	-0.485237000
H	-2.459767000	-4.924765000	1.363614000
H	2.440367000	1.819921000	-0.063288000
H	4.518473000	4.058455000	-2.335627000
H	4.725254000	2.719116000	-1.198967000
H	4.135520000	4.255845000	0.706949000
H	3.912297000	5.578946000	-0.434133000
H	6.329278000	5.404681000	-1.110524000
H	6.555414000	4.076088000	0.017215000
H	5.960246000	5.596462000	1.928664000
H	5.728489000	6.923572000	0.801923000
H	8.137487000	6.752231000	0.102279000
H	8.370909000	5.423417000	1.226472000
H	7.779881000	6.940574000	3.143235000
H	7.543721000	8.268951000	2.019593000
H	9.951617000	8.101086000	1.313619000
H	10.188684000	6.771625000	2.435872000
H	9.600334000	8.286993000	4.355852000
H	9.361844000	9.616355000	3.233883000
H	11.767429000	9.449295000	2.524746000
H	12.006181000	8.120174000	3.645282000
H	11.212512000	10.985243000	4.454847000
H	12.818239000	10.268213000	4.645599000
H	11.453485000	9.646451000	5.583592000
C	0.024629000	-4.379933000	0.741882000
C	1.348200000	-4.212125000	1.120008000
N	1.882426000	-5.455308000	1.080602000
N	0.956794000	-6.358384000	0.694536000
N	-0.163361000	-5.717160000	0.496707000
C	-4.050933000	3.348710000	-0.533735000
N	-3.864077000	4.682662000	-0.267717000
N	-4.984586000	5.325032000	-0.452697000

N	-5.912098000	4.423792000	-0.843839000
C	-5.374144000	3.183652000	-0.912078000
C	-4.764525000	-3.681961000	1.473406000
C	-6.047363000	-3.499773000	0.981536000
N	-6.806861000	-4.397944000	1.650983000
N	-6.052799000	-5.106222000	2.520118000
N	-4.823480000	-4.682224000	2.412023000
C	3.262418000	-5.874058000	1.327043000
C	4.148772000	-5.754153000	0.082493000
C	5.583476000	-6.223951000	0.351014000
C	6.488321000	-6.123641000	-0.883373000
C	7.923986000	-6.597068000	-0.625528000
C	8.829416000	-6.503159000	-1.859780000
C	10.264757000	-6.979315000	-1.604621000
C	11.170091000	-6.888776000	-2.839000000
C	12.605230000	-7.366241000	-2.584931000
C	13.502305000	-7.273283000	-3.822956000
C	-7.273393000	4.859597000	-1.165456000
C	-7.586551000	4.788916000	-2.647276000
C	-8.689953000	4.060006000	-3.101368000
C	-8.996407000	4.014149000	-4.463818000
C	-8.195390000	4.692548000	-5.382355000
C	-7.088504000	5.420155000	-4.934738000
C	-6.786996000	5.470543000	-3.574990000
C	-8.249658000	-4.636219000	1.561060000
C	-9.076467000	-3.492354000	2.114726000
C	-8.900007000	-3.065072000	3.438058000
C	-9.673697000	-2.025507000	3.951130000
C	-10.636228000	-1.404449000	3.149318000
C	-10.818340000	-1.826610000	1.832378000
C	-10.038063000	-2.864815000	1.316790000
H	1.913833000	-3.351818000	1.439268000
H	-5.938747000	2.327743000	-1.244106000
H	-6.453912000	-2.852255000	0.221974000
H	3.209753000	-6.910114000	1.669073000
H	3.651166000	-5.265702000	2.148614000
H	4.154580000	-4.710871000	-0.257660000
H	3.702715000	-6.348495000	-0.723738000
H	5.565133000	-7.264164000	0.704232000
H	6.015894000	-5.630439000	1.168732000
H	6.506135000	-5.082973000	-1.236624000
H	6.051701000	-6.714896000	-1.700254000
H	7.903804000	-7.636639000	-0.268903000
H	8.360559000	-6.004576000	0.191165000

H	8.850085000	-5.463385000	-2.216375000
H	8.391047000	-7.094595000	-2.676175000
H	10.243239000	-8.018559000	-1.246565000
H	10.703692000	-6.387155000	-0.788808000
H	11.193075000	-5.849571000	-3.197643000
H	10.731539000	-7.480682000	-3.655266000
H	12.582200000	-8.404549000	-2.226867000
H	13.043836000	-6.774485000	-1.769842000
H	13.107947000	-7.882810000	-4.644121000
H	14.518329000	-7.621093000	-3.609160000
H	13.573183000	-6.240764000	-4.183910000
H	-7.336690000	5.885123000	-0.793239000
H	-7.975610000	4.248675000	-0.592593000
H	-9.314492000	3.528192000	-2.388121000
H	-9.856474000	3.445432000	-4.804480000
H	-8.429805000	4.655460000	-6.441973000
H	-6.461836000	5.950201000	-5.645676000
H	-5.926280000	6.036029000	-3.228565000
H	-8.418957000	-5.558905000	2.121521000
H	-8.503208000	-4.825624000	0.514848000
H	-8.153114000	-3.547670000	4.062552000
H	-9.529072000	-1.701623000	4.977450000
H	-11.240082000	-0.596419000	3.551321000
H	-11.563509000	-1.348487000	1.203598000
H	-10.180820000	-3.190293000	0.289583000
